# Unique cysteine-enriched, D2L5 and D4L6 extracellular loops in Ca_V_3 T-type channels alter the passage and block of monovalent and divalent ions

**DOI:** 10.1038/s41598-020-69197-3

**Published:** 2020-07-24

**Authors:** Wendy Guan, Robert F. Stephens, Omar Mourad, Amrit Mehta, Julia Fux, J. David Spafford

**Affiliations:** B1-173, Department of Biology, University of Waterloo, Waterloo, ON N2L 3G1 Canada

**Keywords:** Neurophysiology, Permeation and transport, Patch clamp, Voltage clamp, Molecular evolution, Calcium channels, Cardiovascular biology

## Abstract

Invertebrate LCa_V_3 shares the quintessential features of vertebrate Ca_V_3 T-type channels, with a low threshold of channel activation, rapid activation and inactivation kinetics and slow deactivation kinetics compared to other known Ca^2+^ channels, the Ca_V_1 and Ca_V_2 channels. Unlike the vertebrates though, Ca_V_3 T-type channels in non-cnidarian invertebrates possess an alternative exon 12 spanning the D2L5 extracellular loop, which alters the invertebrate LCa_V_3 channel into a higher Na^+^ and lower Ca^2+^ current passing channel, more resembling a classical Na_V_1 Na^+^ channel. Cnidarian Ca_V_3 T-type channels can possess genes with alternative cysteine-rich, D4L6 extracellular loops in a manner reminiscent of the alternative cysteine-rich, D2L5 extracellular loops of non-cnidarian invertebrates. We illustrate here that the preferences for greater Na^+^ or Ca^2+^ ion current passing through Ca_V_3 T-type channels are contributed by paired cysteines within D2L5 and D4L6 extracellular loops looming above the pore selectivity filter. Swapping of invertebrate tri- and tetra-cysteine containing extracellular loops, generates higher Na^+^ current passing channels in human Ca_V_3.2 channels, while corresponding mono- and di-cysteine loop pairs in human Ca_V_3.2 generates greater Ca^2+^ current passing, invertebrate LCa_V_3 channels. Alanine substitutions of unique D2L5 loop cysteines of LCa_V_3 channels increases relative monovalent ion current sizes and increases the potency of Zn^2+^ and Ni^2+^ block by ~ 50× and ~ 10× in loop cysteine mutated channels respectively, acquiring characteristics of the high affinity block of Ca_V_3.2 channels, including the loss of the slowing of inactivation kinetics during Zn^2+^ block. Charge neutralization of a ubiquitous aspartate residue of calcium passing Ca_V_1, Ca_V_2 and Ca_V_3 channels, in the outer pore of the selectivity filter residues in Domain II generates higher Na^+^ current passing channels in a manner that may resemble how the unique D2L5 extracellular loops of invertebrate Ca_V_3 channels may confer a relatively higher peak current size for Na^+^ ions over Ca^2+^ The extracellular loops of Ca_V_3 channels are not engaged with accessory subunit binding, as the other Na^+^ (Na_V_1) and Ca^2+^ (Ca_V_1/Ca_V_2) channels, enabling diversity and expansion of cysteine-bonded extracellular loops, which appears to serve, amongst other possibilities, to alter to the preferences for passage of Ca^2+^ or Na^+^ ions through invertebrate Ca_V_3 channels.

## Introduction

Ca_V_3 T-type channels pass low voltage-activated, inward currents that contribute to pace-making in the mammalian cardiovascular system^1,2^, and to rhythmic spikes, such as low threshold Ca^2+^ potentials (LTCPs) in the thalamus^3^, a region of highest expression in the mammalian brain^4,5^. The most common T-type channel isoforms expressed both in the mammalian heart and brain are Ca_V_3.1 and Ca_V_3.2, which generate rapid, transient and mostly Ca^2+^-selective ionic currents^6^.

We had previously expressed the first non-mammalian Ca_V_3 T-type channel, LCa_V_3, which is a singleton gene derived from the pond snail, *Lymnaea stagnalis*^7,8^. The invertebrate Ca_V_3 homolog shares in key biophysical features of mammalian counterparts, including a capacity to generate rapid and brief inward currents elicited by inhibitory input delivered by hyperpolarization which removes the channel refractoriness due to their inactivation at resting potentials^7^. The invertebrate LCa_V_3 homolog also possesses a “*window current*” of open channels from the significantly overlapping activation and inactivation curves at resting potentials^7^. Invertebrate LCa_V_3 also possesses the typical T-type channel’s characteristically slow rate of deactivation which allows for a significant current influx, even when voltage changes are compelling LCa_V_3 channels to close^7^. A critical difference from vertebrate Ca_V_3 T-type channels was encountered after in vitro expression of an unusual, alternative splice isoform spanning exon 12, which generated high Na^+^ current passing T-type channels^9^. This splice isoform expresses as the only isoform in the invertebrate heart^9^. High Na^+^ current passing T-type channels in invertebrates are generated by swapping of unique extracellular loops rising above the pore’s signature, ion selectivity filter. We first evaluate the importance of the unique pattern of multiple cysteines in the variable extracellular loop in Domain II, by substitution of the cysteines in D2L5 extracellular loop with alanine residues. We observe that Δcys mutations in extracellular D2L5 loops possess a greater monovalent ion current size, and also alters the relative passage of divalent ion currents (Ba^2+^, Sr^2+^) compared to Ca^2+^ and the relative block by Ca^2+^ and other divalent ions (Ni^2+^, Zn^2+^). We then swap extracellular loops between snail LCa_V_3 and mammalian Ca_V_3 channels to illustrate that a mostly Na^+^ or Ca^2+^ current passing T-type channel can be engendered with opposing pairs of extracellular loops containing specific patterning of cysteines that number 1 or 3 or 5 cysteines in Domains II, or 2 and 4 cysteines in Domain IV. What started as an examination of curious T-type Na^+^ currents within invertebrates has led to the discovery of the influences of cysteine-enriched extracellular loops contributing to a unique structure above the channel pore in the regulation of passage of altered Na^+^ or Ca^2+^ permeation and blockade through Ca_V_3 T-type channels.

## Results

### A variable sized Na^+^ current is a distinguishing feature of T-type channels

**Figure 1:**
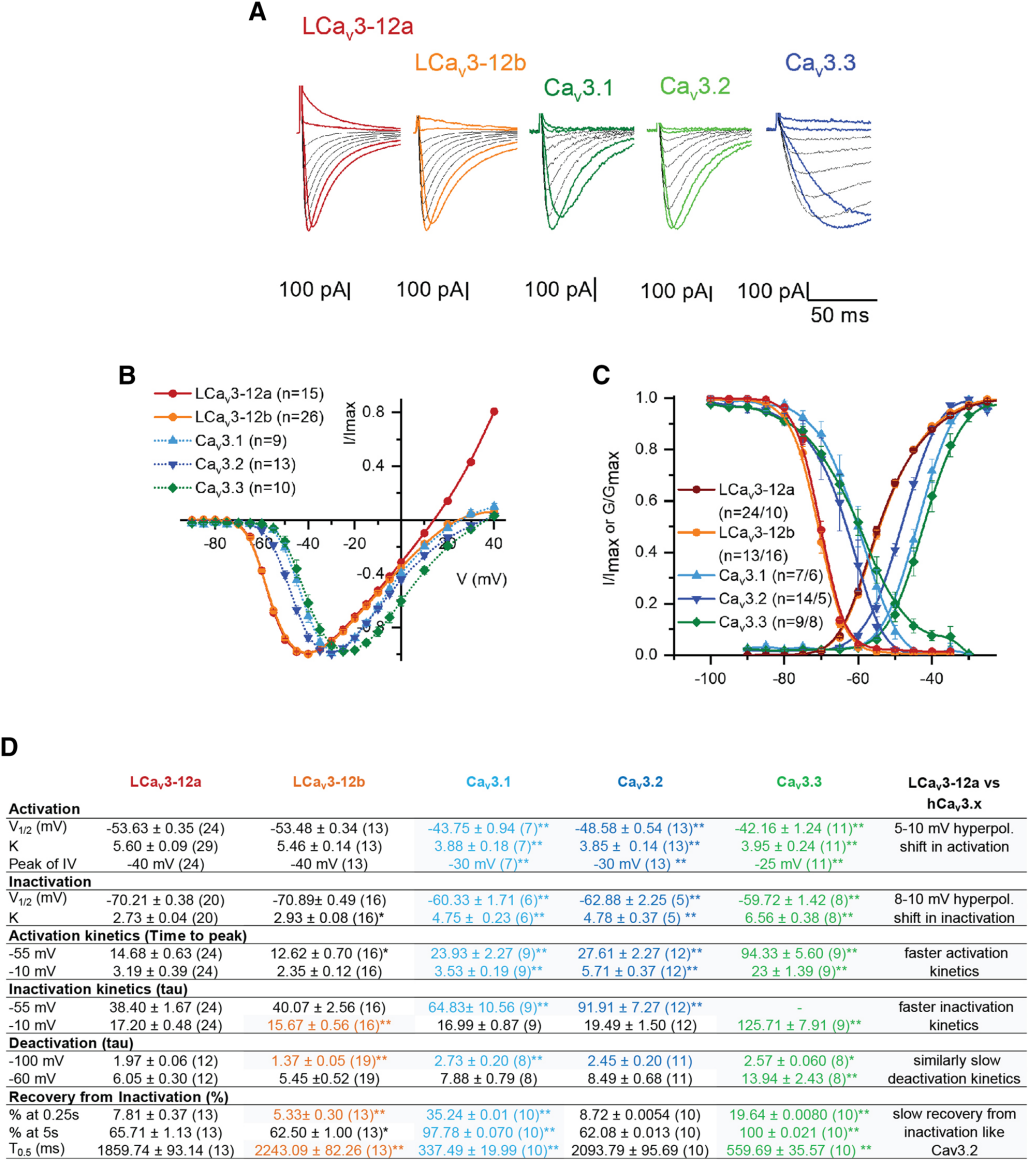
Expressed snail LCa_v_3 channels have more hyperpolarized voltage-sensitivities and faster kinetics than human Ca_v_3 T-type Ca^2+^ channels. The more Na^+^ current passing isoform of snail LCa_v_3 with exon 12a does not vary in biophysical properties compared to more Ca^2+^ current passing isoform with exon 12b. (**A**) Representative Ca_v_3 currents generated from −110 mV to voltage steps from near peak (− 40 to − 30 mV) in 10 mV steps to beyond the reversal potential. (**B**) Current voltage relationships. (**C**) Activation and inactivation curves. (**D**) Comparison of biophysical parameters. Statistics comparison with LCa_V_3–12a using one-way ANOVA combined with a Student-Newman Keuls post hoc test with *p < 0.05, **p < 0.01. Data are represented as mean ± SEM. Grey shaded values represent statistically significant differences between snail LCa_V_3–12a channels. Table 1 provides more detailed statistical comparisons. Color coding of differing Ca_v_3 channels: Ca_v_3.1 (light blue), Ca_v_3.2 (dark blue), Ca_v_3.3 (green), LCa_V_3–12b (orange) and LCa_V_3–12a (red). Data contained in this figure were analyzed and illustrated using OriginPro 2018 (64-bit) SR1 b9.5.1.195.

**Figure 2:**
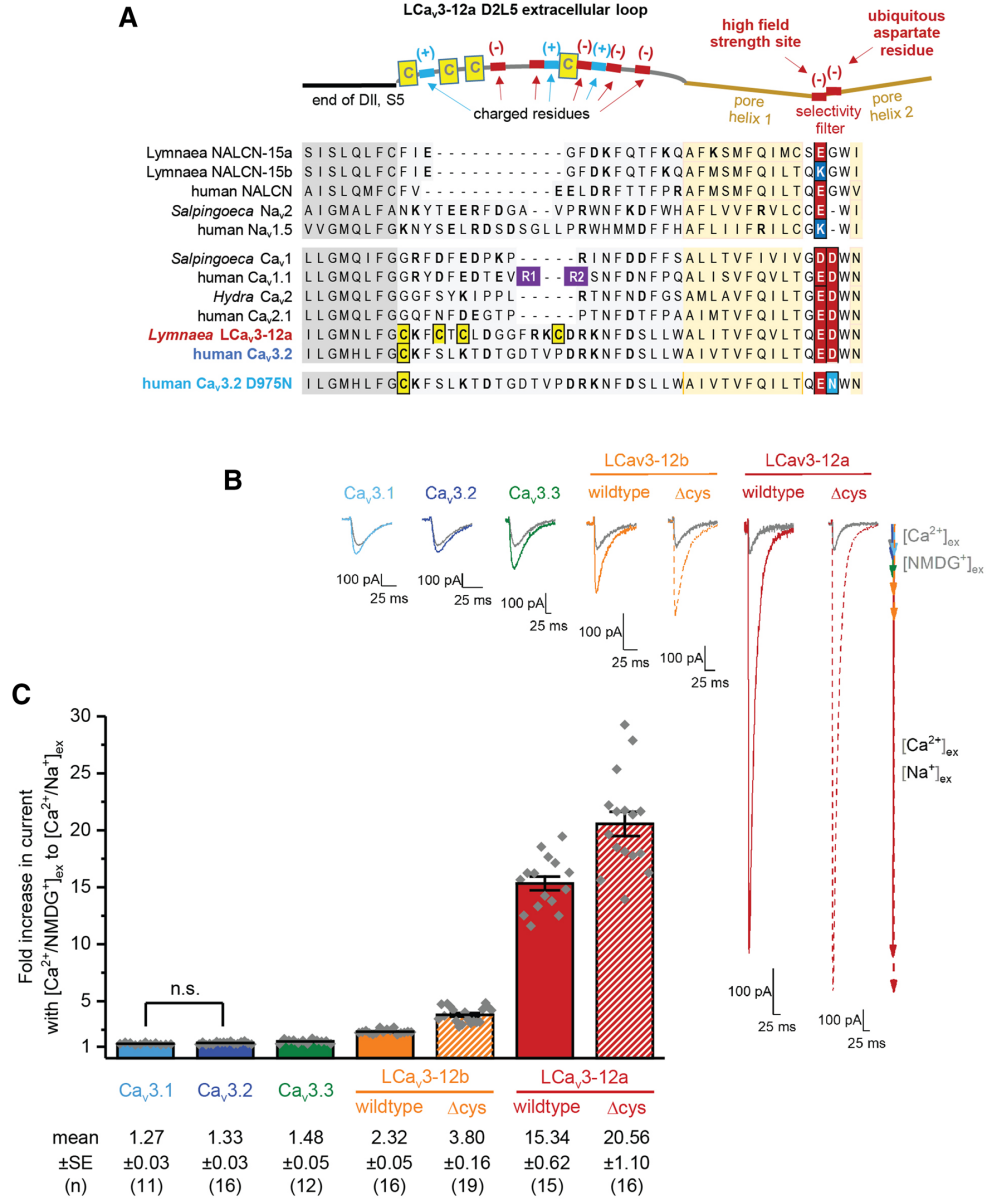
All Ca_v_3 T-type channels will pass Na^+^ in the presence of external Ca^2+^ at physiological concentrations to some degree, but the almost exclusive Na^+^ current passing through snail LCa_v_3 channels requires a unique exon configuration and cysteine content spanning the D2L5 extracellular loop. (**A**) Amino acid alignment (including invertebrate Ca_v_3 and human Ca_v_3.x channels) illustrating the Domain II, L5 extracellular loop and descending helix sequences altered in snail LCa_v_3 channels spanning exon 12a and exon 12b, and highlighting of 3 and 4 cysteine to alanine replacements for LCa_V_3–12a(Δcys) and LCa_V_3–12b(Δcys) mutants. (**B**) Representative traces and (**C**) graph illustrating the dramatic increase in peak current sizes in presence of external Na^+^ ions (Ca^2+^ and Na^+^ external solution) compared to when large weakly permeant monovalent ion, NMDG^+^ replaces Na^+^ ions (Ca^2+^ and NMDG^+^ external solution). Larger size of T-type currents in Δcys mutants suggests that disulphide bonds in extracellular loops is a contributor to Na^+^ or Ca^2+^ current passage through T-type channels. The graph contains mean ± SEM with data replicates (n) illustrated as grey diamonds. Data to generate graphs were compared in a parametric one-way ANOVA with Turkey post hoc test for statistical significance. Statistical significances are tabulated in Supplementary Tables S4 and S5. Data are significant (p < 0.05) unless stated, where n.s. = non-significant. Color coding of differing Ca_v_3 channels: Ca_v_3.1 (light blue), Ca_v_3.2 (dark blue), Ca_v_3.3 (green), LCa_V_3–12b (orange), LCa_V_3–12a (red), LCa_v_3 Δcys mutants (striped orange or red bars, or dotted lines). Electrophysiology data contained in this figure were analyzed and illustrated using OriginPro 2018 (64-bit) SR1 b9.5.1.195. The alignment in Fig. 2a was created using MUltiple Sequence Comparison by Log- Expectation (MUSCLE) at website: https://www.ebi.ac.uk/Tools/msa/muscle/^25^.

The T-type Ca^2+^ channel homolog, LCa_V_3 from sample invertebrate species, pond snail *Lymnaea stagnalis*, shares the quintessential features of human Ca_V_3.1, Ca_V_3.2 and Ca_V_3.3 channels, including a low threshold for channel activation below a typical resting membrane potential (− 65 mV), rapid kinetics and slow de-activation kinetics compared to most other Ca_V_1.x and Ca_V_2.x channels (Fig. 1). Snail channels are notably different in possessing a 5–10 mV hyperpolarized operating range of voltage-sensitivity and possess faster activation and inactivation kinetics compared to the human T-type channels (Fig. 1, Supplementary Table S1). A more remarkable difference in the invertebrate T-type channel homolog was first observed as a strikingly large size of outward currents carried by internal Cs^2+^ ions (see sample current traces, Fig. 1a). Cs^+^ is normally present in standard recording solutions to block contaminating K^+^ currents. The relative contribution of the inward Na^+^ current can be estimated as the fold change in current size in physiological external, 2 mM [Ca^2+^]ex, when an equimolar quantity of 135 mM [Na^+^]ex replaces weakly permeant monovalent ion, (*N*-methyl-d-glucamine) [NMDG^+^]ex (Fig. 2). Native splicing of a novel peptide fragment spanning the extracellular loop before the pore selectivity filter (known as L5 or S5-P) in Domain II of the four domain channel (Fig. 2a) generates large sized LCa_V_3 channel currents with exon 12a that is ~ 15 fold higher peak current size when [Na^+^]ex replaces weakly permeant monovalent ion [NMDG^+^]ex in the presence of [Ca^2+^]ex, compared to the same channel with exon 12b where there is approximately equal Na^+^ and Ca^2+^ contributions to the total peak current size (Fig. 2b,c). We have previously shown that exon 12a which engenders a larger sized T-type channel current in the presence of external Na^+^ is the only splice isoform of the singular T-type channel gene of snails expressed in the snail heart^9^. LCa_V_3 mRNA transcripts containing exon 12a is likely the primary source of voltage-dependent Na^+^ current in the absence of expression of LNa_V_1, the singular Na^+^ channel gene transcript within the snail genome, whose expression is absent outside the central nervous system^9^. The high Na^+^ passing current carried through LCa_V_3–12a channel reveal itself in primary cultured snail cardiomyocytes as a low-voltage, activated current which peaks at – 40 to − 35 mV that is separate from the barium conducting and Ca^2+^-selective high voltage-activated current, peaking at 0 to 5 mV in a voltage ramp generated from – 100 to 100 mV over a one second period^9^.

### A conserved framework of cysteine-containing D2L5 loops within Ca_V_3 T-type channels of differing animal groups

The dramatic increase in relative contribution of the Na^+^ current in the whole cell current of LCav3 channels and T-type currents in snail heart cells is engendered by 17 and 28 amino acid changes, respectively between exon 12a and exon 12b, which represents less than 1% of the large ~ 322 kDa Ca_V_3 T-type channel protein (Fig. 2a). The presence of mutually-exclusive, alternative exons 12a and 12b includes most non-vertebrates Ca_V_3 T-type channels, with a pattern of conserved cysteine placement in the L5 (or S5-P) extracellular loop in Domain II (D2L5) that suggest a highly-organized structural framework of cysteine bridge pairs in invertebrate Ca_V_3 channels (Fig. 2a, Supplementary Figures S1, S2). Exon 12b has a penta-cysteine configuration C…CxC…CxC (most protostomes) or CxxC…C…CxC (some nematodes) and is always longer (range 48–55 aa long, average = 52 aa) than exon 12a (Fig. 2a, Supplementary Figures S1, S2). Exon 12a is shorter (38–46 aa long, average = 41 aa) with a nearly invariant tri-cysteine configuration of D2L5 extracellular loop: CxxC…C (Fig. 2a, Supplementary Figures S1, S2). Basal species like single cell choanoflagellates or cnidarians and vertebrate Ca_V_3 channels have short D2L5 loop (average ~ 39 aa) with no or a single cysteine residue^9^, respectively (Fig. 2a, Supplementary Figure S1). Our goal in this research was to explore the importance of the framework of additional cysteines in the D2L5 loop of invertebrate Ca_V_3 channels in altering the relative peak sizes of the relative monovalent ion (Li^+^, Na^+^, K^+^, Cs^+^) currents, divalent ion currents (Ca^2+^, Ba^2+^, Sr^2+^), and current blockade by external Na^+^, Ca^2+^ and other divalent cations (Ni^2+^ and Zn^2+^).

### Loop cysteines regulates the Na^+^ ion dependent current through T-type channels

**Figure 3:**
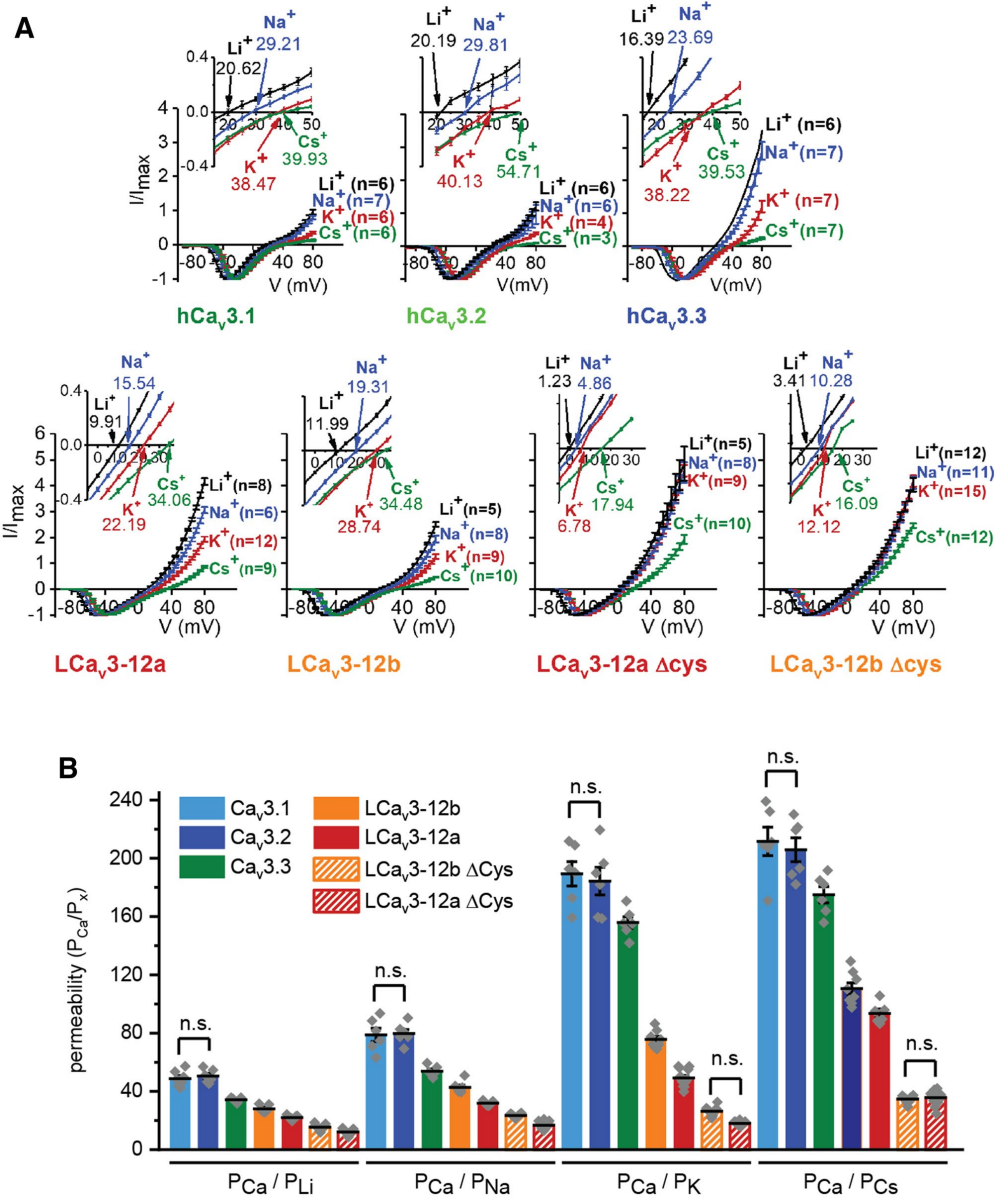
Bi-ionic reversal potentials experiments quantifying the relative monovalent and divalent ion permeation through Ca_v_3 T-type channels by means of measuring the monovalent ion efflux (with 100 mM internal Li^+^ or Na^+^ or K^+^ or Cs^+^ solutions) relative to Ca^2+^ influx (with 4 mM Ca^2+^ external solution). Comparisons are made for human Ca_v_3 channels, snail LCa_v_3 with exons 12a or 12b and LCa_V_3–Δcys mutants where cysteines replace alanines in exon 12. Graphs in (**B**) and (**C**) contain mean ± SEM. with replicates illustrated as grey diamonds. (**A**) Current voltage relationships, with highlights of the currents crossing near the reversal potential (inset). Note the scale of the Y-axis is extended for snail LCa_v_3 channels reflecting the greater monovalent ion permeation compared to human Ca_v_3 channels. (**B**) Shifts in reversal potential reflect a relative permeability (P) change reflected in a PCa/Px ratio, where x is the monovalent ion, Li^+^ or Na^+^ or K^+^ or Cs^+^. Data to generate bar graphs were compared in a parametric one-way ANOVA with statistical significance evaluated in a Turkey post hoc test. Data contained in this figure were analyzed and illustrated using OriginPro 2018 (64-bit) SR1 b9.5.1.195. See Supplementary Tables S2 and S3 for table of mean ± SEM and results of ANOVA analyses, respectively. Data are significant (p < 0.05) unless stated, where n.s. = non-significant. Data for LCa_V_3–12b, LCa_V_3–12a and Ca_v_3.1 in this figure are reproduced integrally from Senatore et al.^9^. Color coding of differing Ca_v_3 channels: Ca_v_3.1 (light blue), Ca_v_3.2 (dark blue), Ca_v_3.3 (green), LCa_V_3–12b (orange), LCa_V_3–12a (red), LCa_v_3 Δcys mutants (striped orange or red bars).

We replaced cysteine residues with alanine residues to convert the tri-cysteine loop of exon 12a and penta-cysteine loop of exon 12b in snail LCa_V_3 channels to superficially resemble the uni-cysteine arrangement in the Domain II L5 loops of vertebrate Ca_V_3 channels (Fig. 2a). Average increases of the peak current size in the presence of external Na^+^ replacing relatively non-permeant monovalent ion, NMDG^+^ was ~ 15 to ~ 20 fold for LCa_V_3–12a (Δcys) and ~ 2.3 to ~ 3.8 fold for LCa_V_3–12b (Δcys) (Fig. 2b,c). The relative permeability differences can be estimated in bi-ionic reversal experiments (Fig. 3), where high [Ca^2+^] in the external solution (4 mM) and a high concentration (100 mM) of monovalent ions (Li^+^, Na^+^, K^+^, Cs^+^) in internal solutions, generates a reversal potential (see inset, Fig. 3a) in a series of current-generating voltage steps that is considered to reflect the relative permeability of Ca^2+^ influx normalized to the permeability for the monovalent ion efflux (Fig. 3b). The calculated relative permeabilities using the bi-ionic method is provided on the bottom of page 34 of Fatt and Ginsborg^10^ based on measurement of reversal potential changes (Fig. 3b). We found the observed rank order from highest to lowest monovalent ion permeability was largely consistent with the observed Na^+^ current contribution to the total size of inward currents (Fig. 2b,c): where LCa_V_3–12a > LCa_V_3–12b > Ca_V_3.3 > Ca_V_3.2 = Ca_V_3.1. An exception to this order is that the outward currents in loop cysteine mutated LCa_V_3–12b (Δcys) channels appear much more permeant to outward monovalent ions (Fig. 3) than expected based on their relative contribution to inward cation currents (Fig. 2b,c).

### Loop cysteines regulate the relative contributions of Na^+^ and Ca^2+^ currents through T-type channels

**Figure 4:**
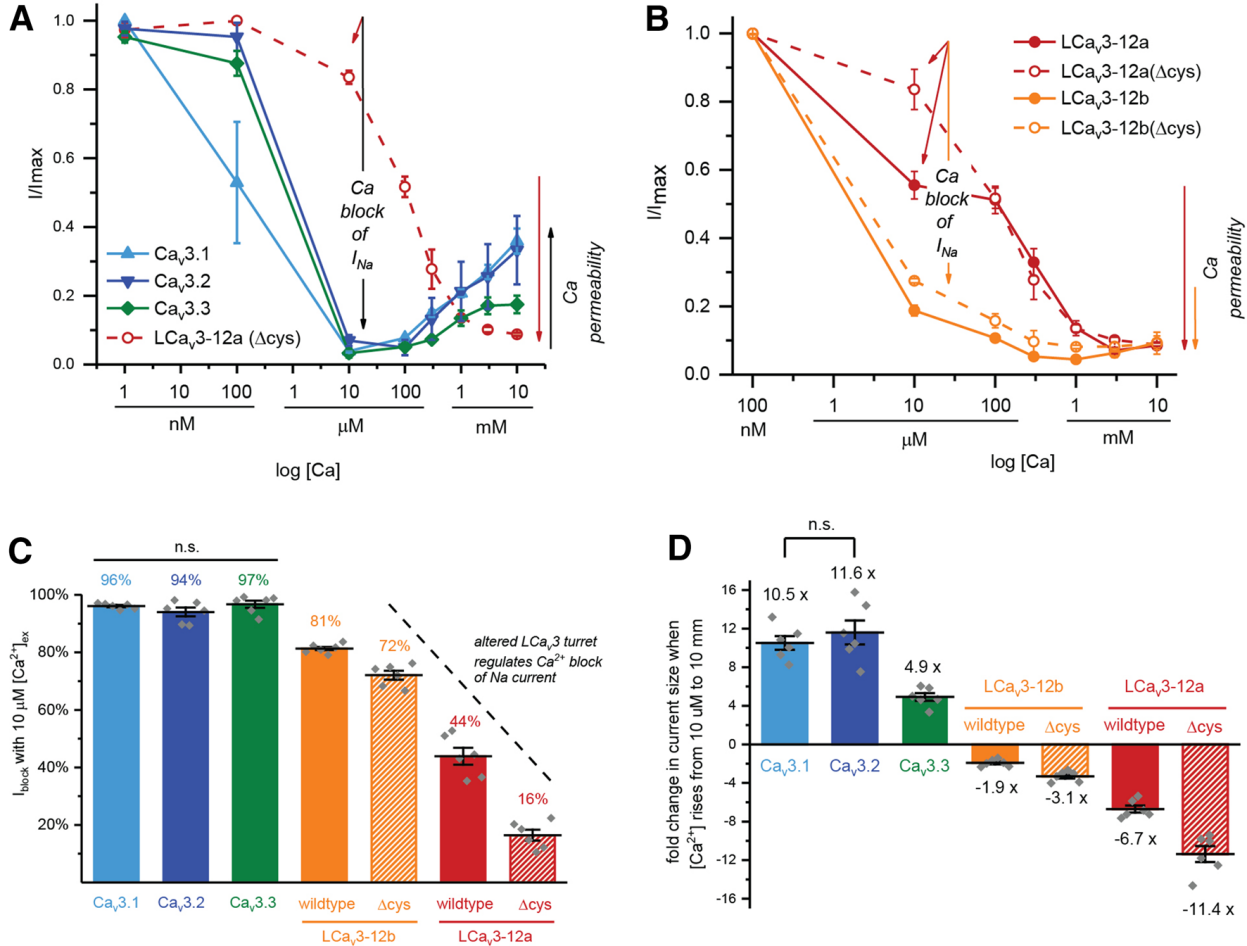
Cysteine-rich D2L5 extracellular loops in LCa_v_3 T-type channels regulate the Ca^2+^ block of the Na^+^ current and relative Ca^2+^ current passage through the physiological range of Ca^2+^ concentrations. Data are represented as mean ± SEM, with statistical comparisons shown in Supplementary Tables S8 and S9. Sample replicate current tracings are shown in Fig. 5. (**A**) Increasing extracellular [Ca^2+^] in presence of 60 mM extracellular [Na^+^] illustrates typical U-shape dependence as Ca^2+^ effectively competes with Na^+^ ions in the pore and passes effectively at physiological mM levels in human Ca_v_3 channels. (**B**) Snail D2L5 extracellular loops regulate an intermediate (LCa_V_3–12b/12bΔcys) to steep (LCa_V_3–12a/12aΔcys) monotonic decline of peak currents with increasing Ca^2+^ concentrations, reflecting the D2L5 extracellular loop’s role in the regulation of the relative size of contributing Na^+^ and Ca^2+^ currents. (**C**) Graph illustrating the weakened capacity of 10 µM external Ca^2+^ to block the inward Na^+^ current through snail LCa_v_3 channels especially in its Δ cys D2L5 extracellular loop mutants. (**D**) Graph illustrating the weakened capacity of snail LCa_v_3 channels, especially its Δ cys D2L5 extracellular loop mutants to pass Ca^2+^ currents at physiological levels of [Ca^2+^] (from 10 µM to 10 mM). Graphs illustrated in (**C**,**D**) contain mean ± SEM with replicates indicated as grey diamonds. Data contained in this figure were analyzed and illustrated using OriginPro 2018 (64-bit) SR1 b9.5.1.195. Data to generate graphs were compared in a parametric one-way ANOVA with statistical significance evaluated in a Turkey post hoc analyses. Data are significant (p < 0.05) unless stated, where *n.s*. non-significant. Data for LCa_V_3–12b, LCa_V_3–12a and Ca_v_3.1 in this figure are reproduced integrally from Senatore et al. 2014^9^. Color coding of differing Ca_v_3 channels: Ca_v_3.1 (light blue), Ca_v_3.2 (dark blue), Ca_v_3.3 (green), LCa_V_3–12b (orange), LCa_V_3–12a (red), LCa_v_3 Δcys mutants (striped orange or red bars), snail LCa_v_3 or human Ca_v_3.2 channels with chimeric extracellular loops (light purple).

We then measured the capacity of increasing extracellular Ca^2+^ doses ranging from 1 × 10^–9^ to 1 × 10^–2^ to compete for passage through the T-type channel pore in the presence of extracellular Na^+^ ions at 60 mM. The voltage of expected peak sized currents shifts with changes in extracellular Ca^2+^ dose, so the peak sized current (Supplementary Figures S3, S4) was measured as the largest peak current size resulting from a voltage step to − 65 mV, − 55 mV, − 45 mV and − 35 mV from a − 110 mV holding potential. Representative peak current traces are illustrated in Supplementary Figure S3 and illustrated graphically in Fig. 4. Human Ca_V_3 channels generate a typical U-shaped response curve, where the large Na^+^ passing current largely dissipates (94–97% in the presence of 10 µM of [Ca^2+^]ex, (i.e. the bottom of the “U” in the U shaped curve) as increasing Ca^2+^ blocks the Na^+^ current from conducting through T-type channels (Figs. 4a, 6c). Increasing [Ca^2+^]ex further still, and the current sizes rise again, reflecting the greater ionic current sizes of human Ca_V_3 channels to Ca^2+^ ions than Na^+^ ions at physiological (mM) levels of [Ca^2+^]ex (Fig. 4a,d). A high Ca^2+^ current size through human Ca_V_3 channels appears to be a reflection of a high capacity of 10 µM Ca^2+^ to block the Na^+^ current (Fig. 4c) and the larger sized Ca^2+^ currents in response to physiological (mM) concentrations of external Ca^2+^ (Fig. 4d). Current sizes rise from ~ 5 to ~ 10-fold from Ca_V_3.3 to Ca_V_3.1 channels, respectively in response to increasing [Ca^2+^]ex from 10 µM to 10 mM levels (Fig. 4d) reflecting their rank order in observed Ca^2+^ current contributions (Fig. 2b,c) and relative Ca^2+^ to monovalent ion permeabilities to the whole cell current (Fig. 3a,b). The U-shaped response to increasing Ca^2+^ is considered an indicator that Na^+^ ions effectively compete for the limited cation binding sites as the cations funnel through the human Ca_V_3 pore at low micromolar [Ca^2+^]ex, and where Ca^2+^ will effectively outcompete Na^+^ ions based on their higher relative Ca^2+^ permeability at physiological (mM) levels of external Ca^2+^ (Fig. 4a). A greater contributing Na^+^ current is reflected in a much weakened capacity of Ca^2+^ to block Na^+^ from passing through snail LCa_V_3 channels, especially at the 10 µM level of [Ca^2+^]ex where the almost complete Ca^2+^ block of current through human channels (94–97%), falls to 81% and 44% for LCa_V_3–12b and LCa_V_3–12a channels, respectively, and falls even further to as low as 16% for LCa_V_3–12a Δcys (Fig. 4b). The reduced effectiveness of Ca^2+^ to block the snail Na^+^ current, especially in LCa_V_3–12a with cysteine loop mutations is evidence of an altered preference of the channel for passage of Ca^2+^ and Na^+^ ions. A monotonic and steady decline of current (Fig. 4b) instead of a U-shaped curve with increasing [Ca^2+^]ex doses, reflects the poor Ca^2+^ current passing capabilities through snail LCa_V_3 channels, regardless of which configuration (exon 12a or exon 12b) of D2L5 extracellular loop.

### Relative sizes of ion currents generated by divalent ions (Ca^2+^, Ba^2+^, Sr^2+^) are altered in cysteine mutated Ca_V_3 channels

**Figure 5:**
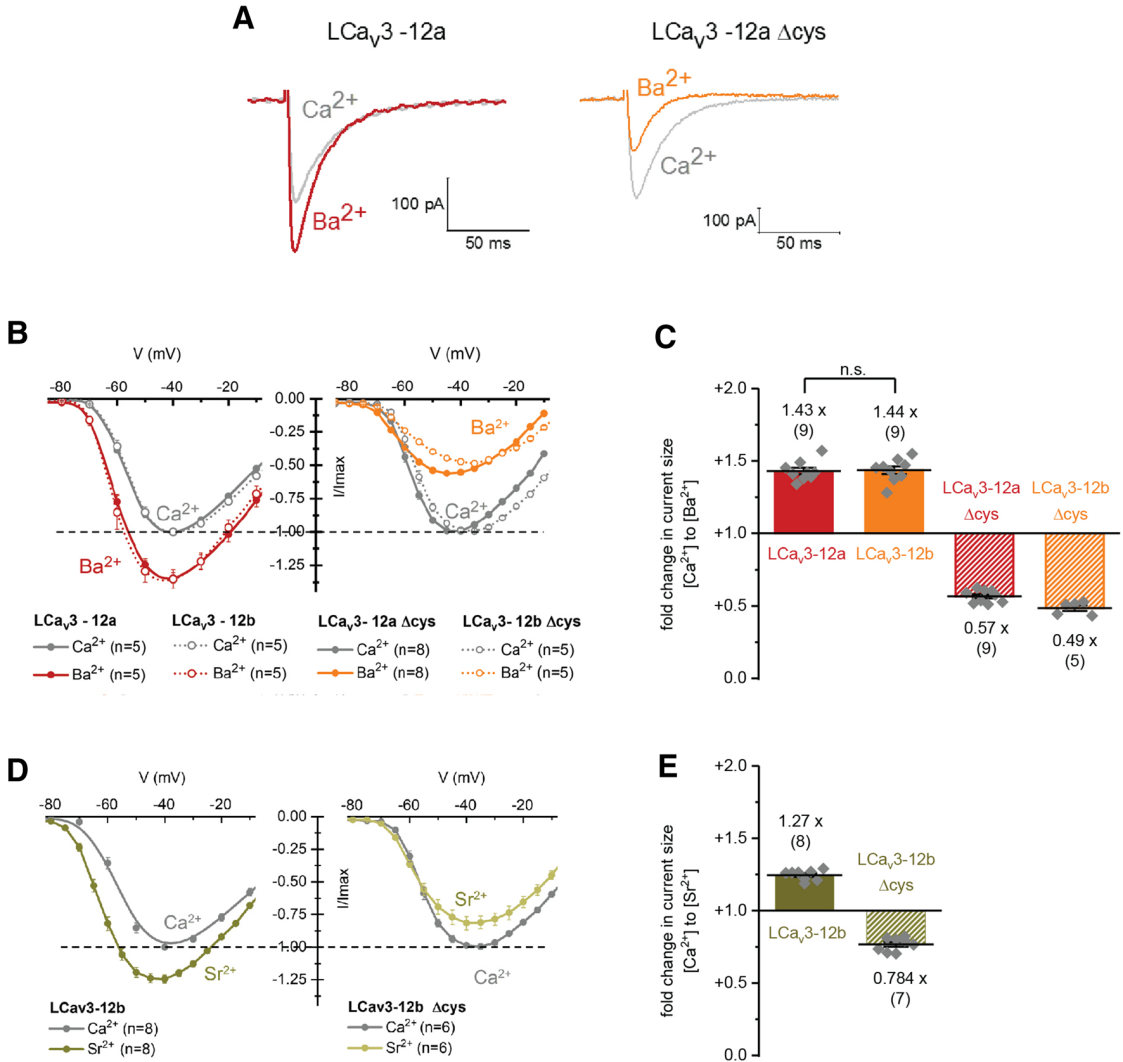
Cysteine replacements with alanines in D2L5 extracellular loops of LCa_v_3 channels alter the relative passage of differing divalent cations, Ba^2+^ or Sr^2+^ relative to Ca^2+^. (**A**) Representative current traces of peak barium (Ba^2+^) and calcium (Ca^2+^) currents normalized to the size of peak Ca^2+^ currents. Current–voltage relationships of the fold change in peak (**B**) Ba^2+^ and (**C**) Sr^2+^ current size normalized to peak Ca^2+^ current levels. Graphs of the fold change in peak current sizes for (**B**) Ba^2+^ and (**C**) Sr^2+^ compared to Ca^2+^ currents. Graphs in (**B**,**C**) are illustrated with mean ± SEM with replicates (n) indicated by grey diamonds. Fold change in LCa_V_3–12a and LCa_V_3–12b T-type channel currents are inverted when cysteines replaces alanines in D2L5 extracellular loops (LCa_v_3 Δcys), where Ca^2+^ currents are larger instead of smaller than Ba^2+^ or Sr^2+^ currents. Data contained in this figure were analyzed and illustrated using OriginPro 2018 (64-bit) SR1 b9.5.1.195.

The uniqueness of Ca^2+^ as a permeant ion through T-type channels can be evaluated when other divalent ions replace Ca^2+^ such as barium (Ba^2+^) (Fig. 5a,b) or strontium (Sr^2+^) (Fig. 5c) as the charge carrier. Native LCa_V_3 channels generate larger peak Ba^2+^ and Sr^2+^ currents (~ 1.4 and ~ 1.3-fold increases, respectively) compared to equivalent Ca^2+^ currents (Fig. 5). Mutations of extracellular loop cysteines disrupts this ratio of relative peak sizes of divalent ion currents in a manner that is consistent with the dramatic current size changes observed for monovalent ions. After Δ cys mutations, the relative sizes of currents are reversed where peak Ca^2+^ and Sr^2+^ currents are now ~ 50 to 80%, respectively of the size of Ba^2+^ currents in LCa_V_3 channels (Fig. 5). Rank order of divalent ion permeabilities does not correspond to the size of ionic radii like the monovalent ions. Ionic radii ^11^ increase with Ca^2+^ (0.99 Ǻ) to Sr^2+^ (1.13Ǻ) to Ba^2+^ (1.35 Ǻ), but amongst different T-type channels, Ca^2+^ ions can generate larger (Ca_V_3.1), smaller (Ca_V_3.2) or no difference in current size (Ca_V_3.3) compared to Ba^2+^ ions^12^.

### Loop cysteines contribute to the block of Ca_V_3 channels by divalent cations (Ni^2+^ and Zn^2+^)

**Figure 6:**
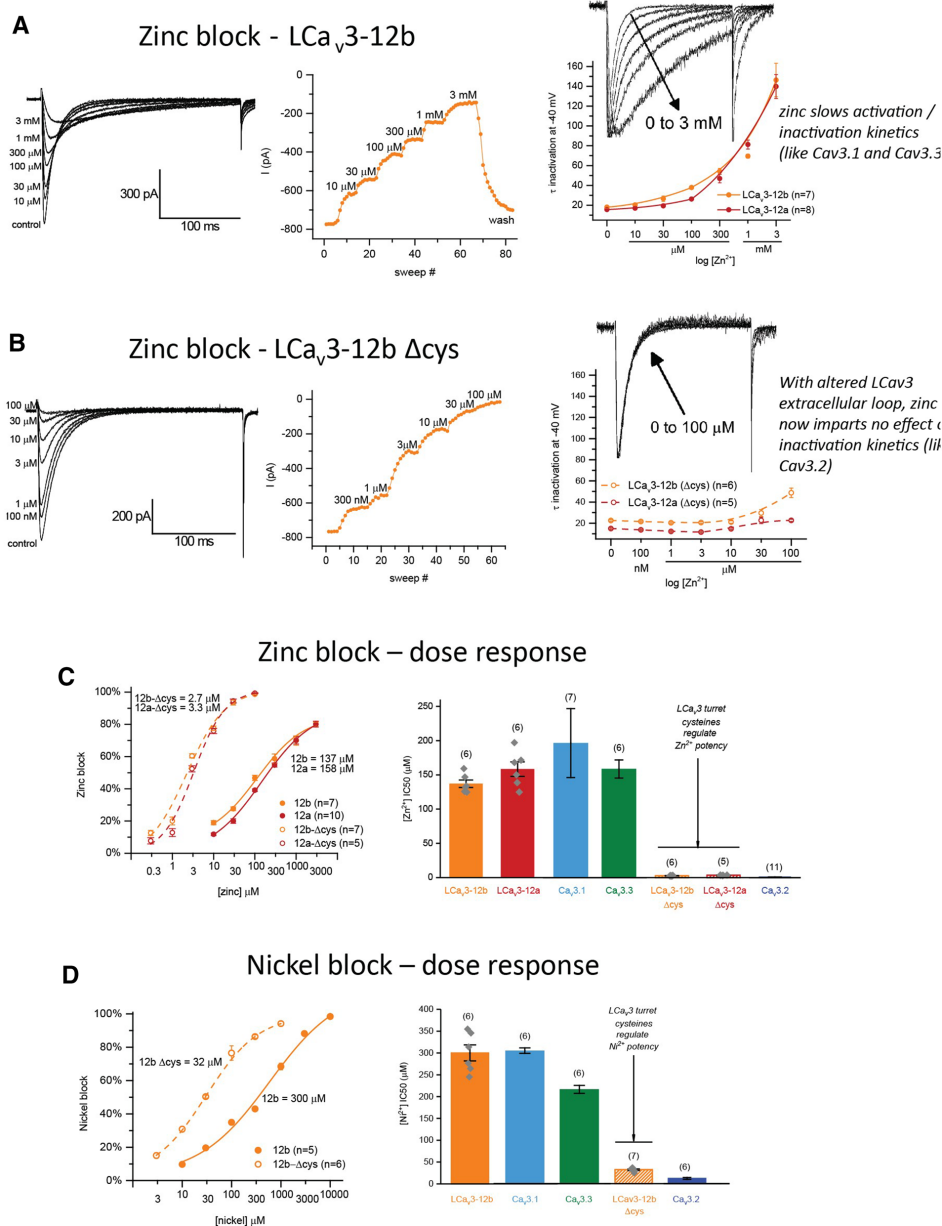
High potency of Zn^2+^ and Ni^2+^ block of Ca_v_3.2 channels is conferred onto LCa_v_3 channels by replacement of cysteines in D2L5 extracellular loops. (**A**,**B**) Representative current traces of LCa_v_3b channels with and without D2L5 extracellular loop cysteine replacements with alanines, in response to Zn^2+^ doses. T-type currents were generated by step depolarizations from − 110 mV to peak voltage (− 40 mV) (left panel). Peak currents per sweep (middle panel) in Ca^2+^ containing external solution (Table 1). Tau mono-exponential fits of mean inactivation kinetics (right panel). Normalized, overlapping currents illustrating kinetic rate differences (right panel, inset). (**C**) Zn^2+^ and (**D**) Ni^2+^ blocking effects. Dose response curves (left panel) and box plot of 50% inhibitory concentrations (IC50) (right panel), with human Ca_v_3.1, Ca_v_3.2 and Ca_v_3.3 shown for comparison. Human Ca_v_3.x channel response to Zn^2+^ taken from^17,18^ and human Ca_v_3.x response to Ni^2+^ taken from^15,16^. The graphs in (**C**,**D**) represent mean ± s.e.m. with replicates illustrated in grey diamonds. IC50 block with Zn^2+^ dose16s of wild type channels (LCa_V_3–12b/LCa_V_3–12a), as well as cysteine mutant pairs (LCa_V_3–12b ΔCys/LCa_V_3–12b ΔCys) are not statistically significant from each other. Snail LCa_v_3 channels possess the weak Zn^2+^ and Ni^2+^ block of Ca_v_3.1 and Ca_v_3.3 channels, but are conferred the high potency of Zn^2+^ and Ni^2+^ block of Ca_v_3.2 channels as well as the characteristic Ca_v_3.2 behavior with a loss of the property where inactivation kinetics slows with increasing Zn^2+^ doses, after alanine replacement of cysteines in D2L5 extracellular loops. Color coding of differing Ca_v_3 channels: Ca_v_3.1 (light blue), Ca_v_3.2 (dark blue), Ca_v_3.3 (green), LCa_V_3–12b (orange), LCa_V_3–12a (red), LCa_v_3 Δcys mutants (striped orange or red bars or dotted lines). Data contained in this figure were analyzed and illustrated using OriginPro 2018 (64-bit) SR1 b9.5.1.195.

T-type channels are mostly resistant to blockade from animal venoms, which contain toxins that specifically target particular ion channels and receptor subtypes, such as specific isoforms of vertebrate Ca^2+^ and Na^+^ channels^13^. The closest subtype specific blocker for T-type channels are divalent ions such as Ni^2+^^14,15^ and Zn^2+^^16,17^ which highly discriminates Ca_V_3.2 over Ca_V_3.1 and Ca_V_3.3 amongst vertebrate T-type channels. Snail LCa_V_3 channels have a 50% Zn^2+^ and Ni^2+^ blocking concentration (IC50 = 140 µM, 300 µM, respectively) that resembles the more weakly blocking Ca_V_3.1 (197 µM, 250 µM) and Ca_V_3.3 (159 µM, 216 µM) channels (Fig. 6).

We addressed whether cysteines in the extracellular loops will regulate the potency of Zn^2+^ and Ni^2+^ block. LCa_V_3 channels increased the 50% blocking concentration of Zn^2+^ and Ni^2+^, ~ 50 and ~ 10-fold in cysteine loop mutated channels, to levels for Zn^2+^ and Ni^2+^ (~ 3 µM, 32 µM) that resemble the high blockade of Ca_V_3.2 channels (0.8 µM, 12 µM), respectively (Fig. 6). The similarities of LCa_V_3 Δcys channels in the blockade of Ca_V_3.2 by Zn^2+^ extends beyond the similarly high potency, to unaltered inactivation kinetics in the presence of Zn^2+^, compared to the dramatic dose-dependent, slowing of inactivation kinetics for Ca_V_3.1 and Ca_V_3.3 channels in the presence of Zn^2+^^16^ (Fig. 6a,b, insets).

### A lowered Ca^2+^ passing preference through invertebrate Ca_V_3 channels containing exon 12a may involve the cysteine-containing D2L5 extracellular loop neutralizing the universal aspartate residue located in the outer pore of the ion selectivity filter

**Figure 7:**
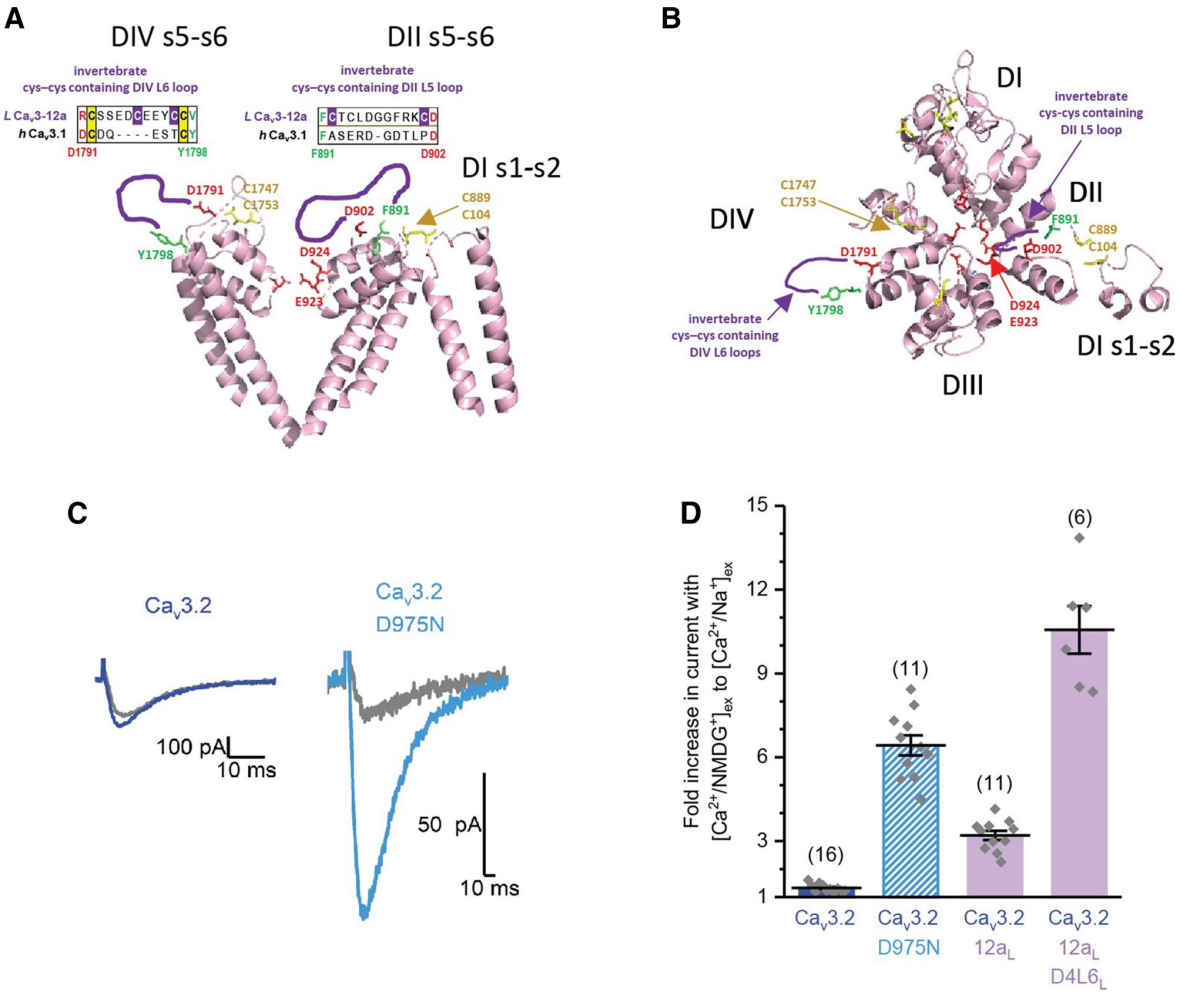
Neutralization of the negatively-charged “calcium beacon” residue in Cav3.2 channels generates high Na^+^ current passing T-type channels. (**A**) Side view and (**B**) top view of human Ca_v_3.1 channel (PDB: 6kzp , 3.1 Å resolution (Zhao et al.^18^), illustrating the opposing DI–DIII and DII–DIV pore loops alone (s5-P-s6), plus DI s1–s2 loop which contains a cysteine bonded to a cysteine in DII L5 (s5-P) loop in Ca_v_3.1. Unique extracellular loops (purple colored lines, sequence above) in DII L5 (s5-P) between F891–D902 and DIV L6 (P-s6) between D1791–Y1798 of Cav3.1 are positioned in invertebrate Cav3 channels. Regions F891–D902 and D1791–Y1798 in Cav3.1 are unresolved in the Cav3.1 structure, indicating that these extracellular loop regions are likely highly flexible in Cav3.1. F891 and D902 are within one amino acid of pore selectivity filter residues (E923 and D924) critical for calcium selectivity. It is modelled that the extracellular loop between cysteines (C1054–C1075) in exon 12a of LCa_v_3 in position between F891–D902 in Ca_v_3.1, brings positively-charged amino acids in proximity of D924, a key aspartate residue omni-present in identified calcium (Ca_v_1, Ca_v_2 and Ca_v_3) channels to date. (**C**) Charge neutralization (D975N) of the “calcium beacon” in Cav3.2T-type channels generates high sodium current passing channels (**D**, sample currents; **E**, graph), revealed as the 6.48 ± 0.98, n = 11 fold increase in peak currents when 135 mM external Na^+^ replacing equimolar impermeant NMDG^+^ in the presence of 2 mM external Ca^2+^. Graph includes mean ± SEM with replicates (n) illustrated with grey diamonds. Large fold increases in sodium current passing channels can similarly be generated in Cav3.2 channels in replacement of D2L5 extracellular loops (Cav3.2-12a) or D2L5/D4L6 extracellular loop pairs (Cav3.2-12a/D4L6) (see Fig. 10). A potential mechanism for the greater Na in LCav3 channels is in the charge neutralization of the calcium beacon, by the juxta-positioning of positively-charged residues within the D2L5 extracellular loop contained within exon 12a. PDB files in Fig. 7a,b are illustrated using PyMOL Molecular Graphics System, Version 2.3, Schrödinger, LLC, https://pymol.org/2/. Data in Fig. 8c,d were analyzed and illustrated using OriginPro 2018 (64-bit) SR1 b9.5.1.195.

So how do the 18 and 27 amino acid differences, respectively between exon 12a and exon 12b generate a starkly different relative Na^+^ and Ca^2+^ current contributions to the whole cell current observed through the large ~ 322 kDa LCa_V_3 T-type channel protein? Both the starting amino acid residues (F891) and terminal amino acid (D902) spanning the unique 9 amino acid cysteine loop of invertebrate exon 12a, is within proximity (~ 1 to 2 amino acids) to D924 (above) and D923 of the ion selectivity filter of the high resolution structure of Ca_V_3.1^18^ (Fig. 7a,b). More than 20% of the non-cysteine residues spanning exon 12 are positively charged (arginine, lysine) or negatively charged amino acids (aspartate, glutamate) (Supplementary Figures S2, S5). The additional cysteine bridge contained in exon 12a may constrains the D2L5 extracellular loop within proximity to influence the pore selectivity filter by electrostatic and/or steric means on key amino acids that alter a preference for passage of Ca^2+^ or Na^+^ currents. The universally conserved aspartate residue of D924 in Ca_V_3.1 is in a key position of the outer pore of the ion selectivity filter found in homologous position in all known Ca^2+^-selective (Ca_V_1, Ca_V_2, and Ca_V_3) channels in the outer pore, and is absent in in all known Na^+^ channels (Na_V_1, Na_V_2) and Na^+^ leak conductance channels (NALCN) (Supplementary Figure S4)^19^. We neutralized D975 in Ca_V_3.2, the equivalent residue in position of D924 in Ca_V_3.1 by replacing charged aspartate (D) residue with a polar asparagine (N) residue. Neutralizing of the charged aspartate (D) residue of the D975N mutation dramatically increased the peak ionic current size observed through Ca_V_3.2 channels in the presence of external Na^+^, in a manner that resembles how the additional D2L5 cysteine loop contained within exon 12a of invertebrate Ca_V_3 channels may neutralize the universal aspartate residue in the outer pore of the ion selective filter of the more Ca^2+^ current passing channels (Fig. 7c,d). We did not observe expressible currents in Ca_V_3.2 channels when this key aspartate residue in the outer pore was substituted with an alanine residue (D975A). The lack of expressible channels with the D975A mutation in Ca_V_3.2 channels, appears to indicate that this key negatively-charged residue in the pore selectivity filter can be neutralized, but will not remain structurally viable if converted to a hydrophobic non-polar residue like alanine.

### D2L5 extracellular loops are not the sole determinant for generating high Na^+^ current passing Ca_V_3 T-type channels in invertebrates

**Figure 8:**
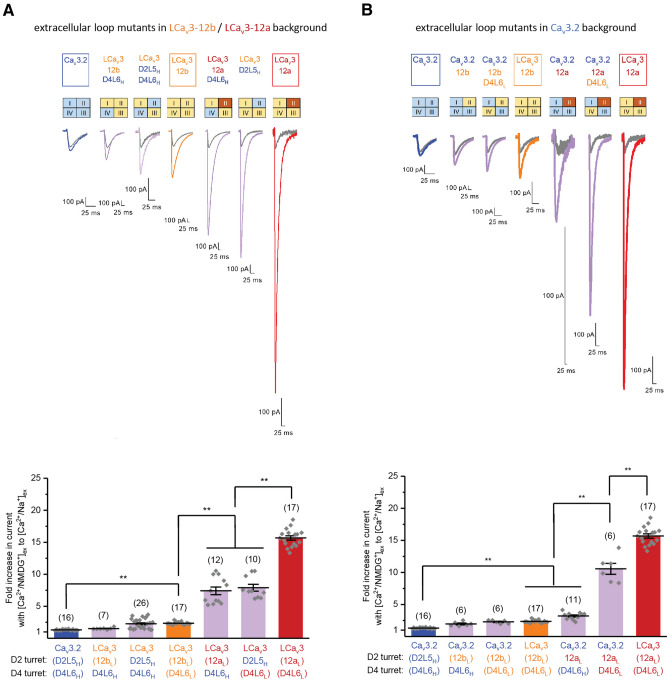
Swapping of snail and human D2L5 and D4L6 extracellular loops can generate high Na^+^ current passing human Ca_v_3.2 channels and high Ca^2+^ current passing snail LCa_v_3 channels. Representative traces (top panels) and graphs (bottom panels) illustrating the fold increases in peak currents when equimolar 135 mM [Na]ex replaces 135 mM [NMDG^+^]ex in presence of 2 mM [Ca^2+^] for D2L5 and D4L6 extracellular loop mutants in (**A**) snail LCa_v_3 (12a/12b) background, and (**B**) human Ca_v_3.2 background. Graphs illustrates mean ± SEM with replicates (n) illustrated by grey diamonds. Data to generate graphs were compared in a parametric one-way ANOVA with statistical significance evaluated in Tukey post hoc analyses. In graphs, data are not significant, unless stated, where **p < 0.01. Statistical significances for (**A**,**B**) are illustrated in Supplementary Tables S4 and S5. Color coding of differing Ca_v_3 channels: Ca_v_3.1 (light blue), Ca_v_3.2 (dark blue), Ca_v_3.3 (green), LCa_V_3–12b (orange), LCa_V_3–12a (red), LCa_v_3 Δcys mutants (striped orange or red bars), snail LCa_v_3 or human Ca_v_3.2 channels with chimeric extracellular loops (light purple). Data contained in this figure were analyzed and illustrated using OriginPro 2018 (64-bit) SR1 b9.5.1.195.

Next, we addressed whether the extracellular D2L5 loop alone is the sole determinant to engender a mostly Ca^2+^ passing current through human Ca_V_3.1 and Ca_V_3.2 channels and a mostly Na^+^ passing current of snail LCa_V_3 channels with exon 12a (Fig. 2b,c). We swapped snail D2L5 loops (spanning snail exons 12a and 12b) and the equivalent D2L5H loop region from human Ca_V_3.2 channels onto human and snail Ca_V_3 channel backgrounds. We found that swapped D2L5 loops in chimeric channels, in both backgrounds, that is LCa_V_3 D2L5H (Fig. 8a) and Ca_V_3.2-12a (Fig. 8b), contribute to an intermediate preference between a mostly Ca^2+^ passing current of human Ca_V_3.2 and the more Na^+^ passing current of snail LCa_V_3–12a channels. These findings suggest that regions other than D2L5 extracellular loops are likely to be contributing to the differences in relative contributions of Na^+^ and Ca^2+^ currents in Ca_V_3 channels.

### Evaluation of the opposing pairs of D2L5 and D4L6 extracellular loops to sizes of relative Na^+^ and Ca^2+^ currents through Ca_V_3 channels

We evaluated the individual contributions of differing D4L6 extracellular loops that we discovered in the two different Ca_V_3 genes containing 2 or 4 cysteines in many anthozoan and scyphozoan cnidarian species found in available genome and transcriptome databases, and the alternative spliced D2L5 loops of other invertebrate Ca_V_3 channels using different combinations of extracellular loop swaps in snail LCa_V_3 and human Ca_V_3.2 channel backgrounds (see Fig. 8). The observed changes in relative contribution of Na^+^ and Ca^2+^ currents in these chimeric channels are dramatic, despite the small size of loop regions of D2L5 (24 and 35 amino acids) and D4L6 extracellular loops (16 and 19 amino acids) that were swapped compared to the total channel length of 2,353 and 2,685 amino acids for full-length Ca_V_3.2 and LCa_V_3 channels, respectively.

### Generating mostly Ca^2+^ current passing human Ca_V_3.2-like channels in snail LCa_V_3 background

The relative Na^+^ ion contribution to the whole cell currents (illustrated in brackets below) was evaluated as the fold increase in current size in presence of 2 mM external Ca^2+^ when equimolar 135 mM external Na^+^ replaces 135 mM larger monovalent ion, NMDG^+^ (as illustrated in Fig. 2b,c). We attempted to transform snail LCa_V_3–12a which has the highest relative contribution of Na^+^ ions to the measured whole cell current (15.68 ± 0.34, n = 15) to resemble the high Ca^2+^ current passing channel, human Ca_V_3.2 (1.31 ± 0.025, n = 16) (Fig. 8a).

Replacement of snail LCa_V_3 with either D2L5H or D4L6H loops of human Ca_V_3.2 channels, cut the relative observed Na^+^-dependent, current size through snail LCa_V_3 channels by approximately half (7.42 ± 0.60, n = 12, 7.90 ± 0.54, n = 10) (Fig. 8a). For LCa_V_3 channels to achieve a higher Ca^2+^ passing channel approximating to the high Ca^2+^ passing of human Ca_V_3.2 channel requires dual (D2L5H and D4L6H) human Ca_V_3.2 loop swaps (2.27 ± 0.15, n = 26) in snail LCa_V_3 (Fig. 8a). The highest Ca^2+^ current contributing snail channel isoform with exon 12b (2.36 ± 0.054, n = 17) bears a longer penta-cysteine loop of 37 amino acids and appears to be the invertebrate equivalent of the native human Ca_V_3.2 channel (Fig. 2a, Supplementary Figures S1, S2) in its passing of a high Ca^2+^ current of the total whole cell current. LCa_V_3–12b channels with the human D4L6H loop are no different statistically in the relative contribution of Ca^2+^ to the whole cell current (1.52 ± 0.060, n = 7) as the mostly Ca^2+^ current passing wild-type human Ca_V_3.2 channels (1.31 ± 0.025, n = 16) (Fig. 8a). Taken together, any of D2L5 loops from snail exon 12b or human (D2L5H) with human D4L6H generates a high Ca^2+^ current passing channel (~ 1.52, ~ 2.27) in snail LCa_V_3, that is lost when the snail channel bears (Lymnaea) snail exon 12a D2L5L loop (~ 7.9, ~ 15.7) or if a chimeric snail channel with D2L5H possesses its native D4L5L loop (~ 7.4).

### Generating mostly Na^+^ current passing (snail LCa_V_3–12a like) channels in human Ca_V_3.2 background

We observe a capacity of swapped D2L5 and D4L6 loops in generating a higher Na^+^ passing current in the normally mostly Ca^2+^ current passing, human Ca_V_3.2 channel (Fig. 8b). The Na^+^ contribution to the whole cell current of Ca_V_3.2 channels in the presence of snail exon 12a (3.20 ± 0.17, n = 11), dramatically increases further to the level of the mostly Na^+^ current passing channels of snail LCa_V_3–12a (15.68 ± 0.34, n = 17) in the presence of both snail exon 12a and the snail D4L6L loop (10.56 ± 0.85, n = 6) (Fig. 8b). Placement of snail exon 12b alone (1.96 ± 0.14, n = 6) or combination of exon 12b and the snail D4L6L loop (2.29 ± 0.11, n = 6) in human Ca_V_3.2 channels generates a high Ca^2+^ current passing channels that are not significantly different than wild-type Ca_V_3.2 channels (1.31 ± 0.025, n = 16) (Fig. 8b). Taken together, we observe that a significant peak Na^+^ current through human Ca_V_3 channels (~ 1.3 fold) requires snail exon 12a (~ 3.2 fold) and also the snail D4L6L to manifest the very high peak Na^+^ current contribution through wild-type snail LCa_V_3 channels (~ 10.6 fold , ~ 15.7 fold) (Fig. 8b).

### The relative Ca^2+^ to Li^+^ or Na^+^ permeabilities in bi-ionic recording conditions correspond to the measured Na^+^ contribution to the whole cell current

**Figure 9:**
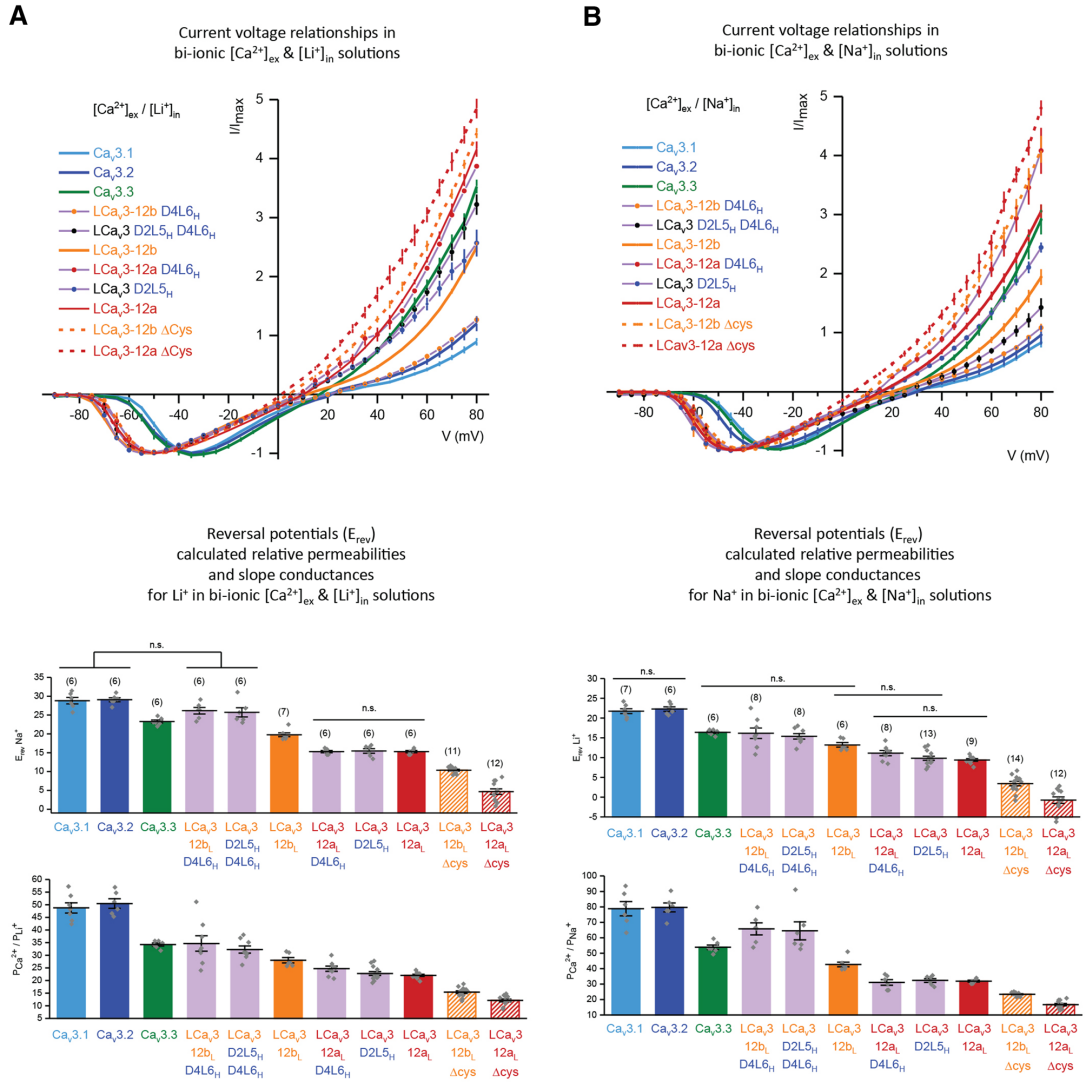
Normalized peak currents in response to voltage steps generated in conditions of extracellular 4 mM [Ca^2+^] and 100 mM intracellular Li^+^ (**A**), or 100 mM intracellular Na^+^ (**B**), with reverse potentials and calculated relative permeabilities shown graphically. Note the differing reversal potentials and slope conductances of outward currents in the current–voltage relationships for (**A**, Li^+^) and (**B**, Na^+^), and a characteristic uniformity of voltage-dependent inward Ca^2+^ currents peaking at more hyperpolarized voltages for wild type and extracellular loop chimeras of snail LCa_v_3 T-type channels, compared to human Ca_v_3.1, Ca_v_3.2 and Ca_v_3.3 channels (see Fig. 1 for biophysical description of wild type snail LCa_v_3 and human Ca_v_3.x channels). Graphs illustrate mean ± SEM with replicates (n) illustrated by grey diamonds. Data to generate graphs were compared in a parametric one-way ANOVA with statistical significance evaluated in Tukey post hoc analyses. Data displayed in graphs are not significant, unless stated, where **p < 0.01. Statistical significances for (**A**,**B**) are illustrated in Supplementary Tables S6 and S7, respectively. Color coding of differing Ca_v_3 channels: Ca_v_3.1 (light blue), Ca_v_3.2 (dark blue), Ca_v_3.3 (green), LCa_V_3–12b (orange), LCa_V_3–12a (red), LCa_v_3 Δcys mutants (striped orange or red bars), snail LCa_v_3 or human Ca_v_3.2 channels with chimeric extracellular loops (light purple). Data contained in this figure were analyzed and illustrated using OriginPro 2018 (64-bit) SR1 b9.5.1.195.

To quantify the relative permeabilities of Ca^2+^ ion to monovalent ion X^+^ (PCa^2+^/PX^+^) in the extracellular loop chimeras, we evaluated the monovalent ion current as an outward current flux, relative to the inward Ca^2+^ influx, generated in bi-ionic conditions where the monovalent ion (Li^+^ or Na^+^) is held at intracellular concentrations at 100 mM in the presence of extracellular divalent cation (Ca^2+^) held at 4 mM (as previously illustrated for wild type and LCa_V_3 Δcys channels in Fig. 3). The relative permeabilities of the Ca^2+^ influx to monovalent ion efflux (PCa^2+^/PLi^+^) (Fig. 9a) and (PCa^2+^/PNa^+^) (Fig. 9b) is a measure based on their influence on the reversal potential calculated in a bi-ionic Nernst potential equation of Fatt and Ginsborg^10^. The degree of relative Na^+^ current passing through the D2L5 and D4L6 loop chimeras largely reflect a rank order based on their calculated relative permeabilities for Li^+^ (Fig. 9a) and Na^+^ (Fig. 9b) in bi-ionic conditions. The calculated relative permeabilities closely correspond to the changes in Na^+^ and Ca^2+^ contributions to the whole cell current measured by the increase in the total inward current size measured in the presence of 135 mM Na^+^ compared to impermeant monovalent ion, NMDG^+^ (Fig. 8a,b). Note the highly variable reversal potentials of outward currents in the current–voltage relationships for (Fig. 9a, Li^+^) and (Fig. 9b, Na^+^), while the voltage-dependent inward Ca^2+^ currents are peaking uniformly at a characteristically (− 20 mV) more hyperpolarized voltages for all wild type and loop chimeras of snail LCa_V_3 T-type channels, compared to human Ca_V_3.1, Ca_V_3.2 and Ca_V_3.3 channels. The trend lines for Li^+^ permeabilities (Fig. 9a) and Na^+^ permeabilities (Fig. 9b) were similar, except Li^+^ is a more permeable ion with a smaller ionic radius than Na^+^. The calculated relative permeabilities confirm that the increasing size of inward currents in the presence of external Na^+^ compared to impermeant monovalent ion NMDG^+^ as a charge carrier, corresponds to a relative increase in monovalent ion permeability through Ca_V_3 T-type channel, compared to relative Ca^2+^ ion permeability.

### A higher Na^+^ permeation through the loop chimeras correspond to a weaker Ca^***2***+^ block of the Na^+^ current at 10 uM of external Ca^2+^

**Figure 10:**
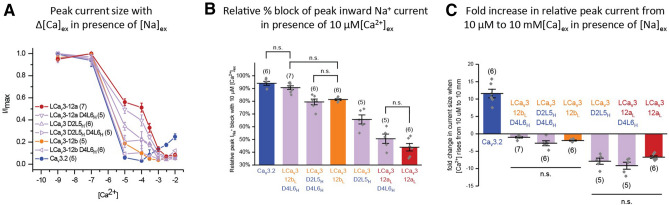
D2L5 and D4L6 extracellular loops regulate the degree of Ca^2+^ block of the Na^+^ current and the fold increase in relative peak Ca^2+^ current size when external Ca^2+^ rises from 10 µM [Ca^2+^]ex to the physiological (mM) range. (**A**) Normalized peak current sizes in response to increasing concentrations of [Ca^2+^]_ex_ from 10–9 to 10–2 M in presence 60 mM [Na^+^]_ex_ for wild type snail LCa_V_3–12a and LCa_V_3–12b channels, and chimeric snail LCa_v_3 channels with D2L5 and D2L6 extracellular loops from human Ca_v_3.2. (**B**) Bar graphs of the normalized peak current blockade at 10 µM [Ca^2+^]_ex_, the maximally effective blocking Ca^2+^ concentration for human Ca_v_3 channels (i.e. bottom of “U” shaped curve in **A**). (**C**) Bar graphs of the fold change in normalized peak currents from 10 µM to 10 mM [Ca^2+^]_ex_. Graphs illustrate mean ± SEM with replicates (n) illustrated as grey diamonds. Data to generate graphs were compared in a parametric one-way ANOVA with a Tukey post hoc analyses to test for statistical significances. Statistical significances are tabulated in Supplementary Tables S8 and S9 for (**B**,**C**). Data are significant (p < 0.01), unless stated, where *n.s*.non-significant. Data for LCa_V_3–12b, LCa_V_3–12a and Ca_v_3.1 in this figure are reproduced integrally from Senatore et al.^9^. Color coding of differing Ca_v_3 channels: Ca_v_3.2 (dark blue), LCa_V_3–12b (orange), LCa_V_3–12a (red), LCa_v_3 Δcys mutants (striped orange or red bars), snail LCa_v_3 or human Ca_v_3.2 channels with chimeric extracellular loops (light purple). Data contained in this figure were analyzed and illustrated using OriginPro 2018 (64-bit) SR1 b9.5.1.195.

The competition between Ca^2+^ and Na^+^ for passage through the Ca_V_3 channel pore of the extracellular loop chimeras can be evaluated by increasing extracellular Ca^2+^ from 1 nM (1 × 10^–9^) to 10 mM (1 × 10^–2^) in the presence of a constant 60 mM external Na^+^ concentration as illustrated previously for wild type and LCa_V_3 Δcys channels in Fig. 4 and Supplementary Figure S3. The voltage of expected peak sized currents shift with changes in extracellular Ca^2+^ dose, so the peak sized current (Fig. 10, Supplementary Figure S7) was measured as the largest current size resulting from a voltage step to − 65 mV, − 55 mV, − 45 mV and − 35 mV from a − 110 mV holding potential. We find that the degree to which Ca_V_3 channels are Na^+^ current passing corresponds to Ca_V_3 channels with a weaker Ca^2+^ block of the Na^+^ current (Fig. 10, Supplementary Figure S7). The chimeric snail Ca^2+^ channel LCa_V_3–12b with the human D4L6H loop, for example is equally Ca^2+^current passing as human Ca_V_3.2 and possesses an equivalently strong (90.63% ± 0.014, n = 7) Ca^2+^ block of the Ca^2+^ current compared to wild-type Ca_V_3.2 (94.03% ± 0.015, n = 6) (Fig. 10, Supplementary Figure S7). The more significantly Na^+^ current passing chimeras of LCa_V_3 correspondingly possess a weaker Ca^2+^ block of the Na^+^ current, such as LCa_V_3–12a/D4L6H (50.59% ± 0.036, n = 5) and LCa_V_3/D2L5H (65.71 ± 0.035, n = 5) (Fig. 10, Supplementary Figure S7).

### The loop chimeras possessing a relatively high Na^+^ current diminishes rather than increase the size of whole cell currents when external Ca^2+^ concentrations rise through the physiological range

The high Na^+^ current passing LCa_V_3 channel with exon 12a exhibits a monotonic decline of current size as extracellular Ca^2+^ increases from 10 µM to 10 mM, as Ca^2+^ is less passing relative to blocking the more permeant Na^+^ ion from passage through the Ca_V_3 channel pore (Fig. 10, Supplementary Figure S7). An equally steep monotonic decline in peak current size is observed with increasing Ca^2+^ concentration from 10 µM to 10 mM for the loop chimeric channels which possess high Na^+^ current passing capabilities such as LCa_V_3–12a/D4L6H and LCa_V_3/D2L5H.

The more Ca^2+^ current generating loop chimeras, on the other hand, such as LCa_V_3–12b/D4L6H and LCa_V_3–D2L5H/D4L6H have a much reduced decline in current size with increasing external Ca^2+^ concentration rises suggesting that these channels are more readily passing Ca^2+^ rather than impeded by the competing Na^+^ (Fig. 10, Supplementary Figure S7).

### Contributions of the differing single channel currents are inferred by evaluation of the recording of the composite whole cell currents

We have measured the relative contribution of Na^+^ and Ca^2+^ currents through differing Ca_V_3 channels including chimeric and mutated channels by different approaches in this manuscript. These include: (a) measuring the relative Na^+^ and Ca^2+^ contribution to peak inward currents, or (b) as relative peak inward Ca^2+^ to peak outward monovalent (X^+^) ionic currents, and their influences on the reversal potential; (c) identifying individual current contributions by means of differing external and internal solutions lacking or containing variable concentrations of Na^+^ and Ca^2+^ ions; (d) comparing the relative contribution to peak currents or blockade with differing divalent ions: Ca^2+^, Ba^2+^, Sr^2+^, Ni^2+^, Zn^2+^; and differing monovalent ions: Cs^+^, K^+^, Na^+^, Li^+^. The differing contributions of single channel conductances for Na^+^ and Ca^2+^ ions would be gained in single channel recording. What we have observed at the whole cell level in this manuscript, nonetheless, is a consistency in the data set reflecting the greater Na^+^ or Ca^2+^ passing character through Ca_V_3 T-type channels, gained in measurement of their observed relative contribution to peak Na^+^ and Ca^2+^ currents, the calculated permeabilities that were generated in bi-ionic solutions, the changing size of relative current contributions with increasing external Ca^2+^ doses in the presence of Na^+^ ions, and the behaviors of differing monovalent or divalent ions other than Na^+^ and Ca^2+^ respectively.

### The differing current densities recorded from transfected HEK-293 T cells was not a significant factor in the assessment of the relative contributions of Na^+^ and Ca^2+^ currents through Ca_V_3 channels

A possible caveat in working with in vitro expressed channels is the observed high variability in the current densities (pA/pF) in replicate recordings from the same or different batches of transfected HEK-293T cells (Supplementary Figure S8), which can contribute to observable differences in channel properties. We observe no apparent influences of the highly variable current densities in individual whole cell recordings, such as the relative pattern of contributing Na^+^ and Ca^2+^ currents to the five wild type channels (Ca_V_3.1, Ca_V_3.2, Ca_V_3.3, LCa_V_3–12b, LCa_V_3–12a) and eight chimeric LCa_V_3 or Ca_V_3.2 channels or two mutated LCa_V_3 Δcys channels (Supplementary Figure S8). Expressed chimeric clones do generate a lower range of average current densities than their wild-type counterparts, for both the human Ca_V_3.2 channels containing snail extracellular loops, and the snail LCa_V_3 channels containing mammalian extracellular loops (Supplementary Figure S9).

The generally lower current densities of chimeric channels could reflect a compromised membrane trafficking and/or expression because of the xeno-graphing of poorly-compatible extracellular loops to native channels. The highest average current densities (i.e. LCa_V_3–12a, LCa_V_3–12a Δcys) are enhanced for Na^+^ passing channels in our use of high 60 mM, 100 mM or 135 mM external [Na^+^] compared to the much lower concentration of external Ca^2+^ of 2 mM used in our experiments (Supplementary Figure S9).

## Discussion

### Why do invertebrate Ca_V_3 T-type channels have alternative spliced isoforms with a high preference for passage of Na^+^ currents?

We illustrate here that a major structural determinant for the greater relative passage of Na^+^ or Ca^2+^ ions through Ca_V_3 T-type channels involves cysteine-rich, extracellular D2L5 and D4L6 loops, which have alternative forms in non-vertebrates (see Supplementary Figure S1). Exon 12a spanning the D2L5 loop contributes to a more Na^+^ current passing isoform of invertebrate Ca_V_3 T-type channel that more resembles the classical Na_V_1 channels. We have recorded LCa_V_3 channels containing exon 12a in vitro and matched the expression phenotype to a corresponding Ni^2+^ and mibefradil sensitive, but 1,4-dihydropyridine insensitive, low voltage activated, Na^+^ current recorded in primary cultured, snail cardiomyocytes ^9^.

Alternative isoforms of exon 12 are present in non-cnidarian invertebrate Ca_V_3 T-type channel genomes to generate alternative mostly Na^+^ current (exon 12a) or more Ca^2+^ current (exon 12b) passing channels. The alternative extracellular loop in Domain II of Ca_V_3 T-type channels make a first appearance in extant relatives of the *Platyhelminthes* (Supplementary Figures S1, S5). *Platyhelminth* (which include the flatworms) are basal multicellular invertebrates with a rudimentary body cavity (pseudo-coelom). While possession of a Na_V_1 channel gene is optional outside of the vertebrates, Ca_V_3 T-type channel genes are ubiquitously found in genomes of every multicellular animal to date outside of basal sponge and ctenophores. We propose that Ca_V_3 T-type channels with exon 12a as an important contributor to Na^+^ influx in lieu of Na_V_1 channels which are lacking often outside the central nervous systems of non-vertebrates.

### Anthozoan and scyphozoan contain alternative D4L6 extracellular loops in their Ca_V_3 channels

We combed through available genome and transcriptome databases to identify alternative extracellular loops varying in sequence, size and patterning of cysteines, resembling the D2L5 loops spanning exon 12a and exon 12b of invertebrate Ca_V_3 channels. The difference is that this evolutionary pathway involves cnidarians and D4L6 loops instead, and a sequence diversity created through gene duplication rather than alternative splicing. Cnidarians are the only non-vertebrates outside of the most basal representatives in single cell choanoflagellates (*Salpingoeca*) and placozoans (*Trichoplax*), without tri-cysteine D2L5 loops (exon 12a) and/or penta-cysteine D2L5 (exon 12b) extracellular loops (Supplementary Figures S1, S2). Instead anthozoan and scyphozoan classes of cnidarians are uniquely endowed amongst non-vertebrates, (besides flatworms), in containing two rather than a singleton Ca_V_3 T-type channel gene in their genome (Supplementary Figures S1, S6). One of the anthozoan and scyphozoan Ca_V_3 genes possesses a shorter di-cysteine (C..C) D4L6 loop of ~ 16 amino acids (Gene A) which resembles the D4L6 loop of the greater Ca^2+^ current passing vertebrate Ca_V_3 channels, while the other anthozoan and scyphozoan bears a longer tetra-cysteine (C..C..CC) D4L6 loop of 19 amino acids (Gene B) resembling the D4L6 loop of the greater Na^+^ current passing invertebrate Ca_V_3 channels (see Supplementary Figure S2 for cartoon illustration of the D2L5 and D4L6 loop configurations, and Supplementary Figures S2, S6, respectively for sample sequences of D2L5 and D4L6 loops). The presence of two additional cysteines in D4L6 extracellular loop found in most invertebrate Ca_V_3 channels, which can generate a high Na^+^ passing T-type channel, lead us to hypothesize that cnidarian Ca_V_3 channels may contribute to alternative high Na^+^ or Ca^2+^ passing T-type channels by means of their two Ca_V_3 genes, as the alternative D2L5 loops possessed by other invertebrate Ca_V_3 channels.

### How is it that Ca_V_3 T-type channels, but not other Ca^2+^ channels (Ca_V_1 or Ca_V_2) channels or Na_V_1 channels can possess alternative extracellular loop forms which alters the preference for passing Na^+^ or Ca^2+^?

**Figure 11:**
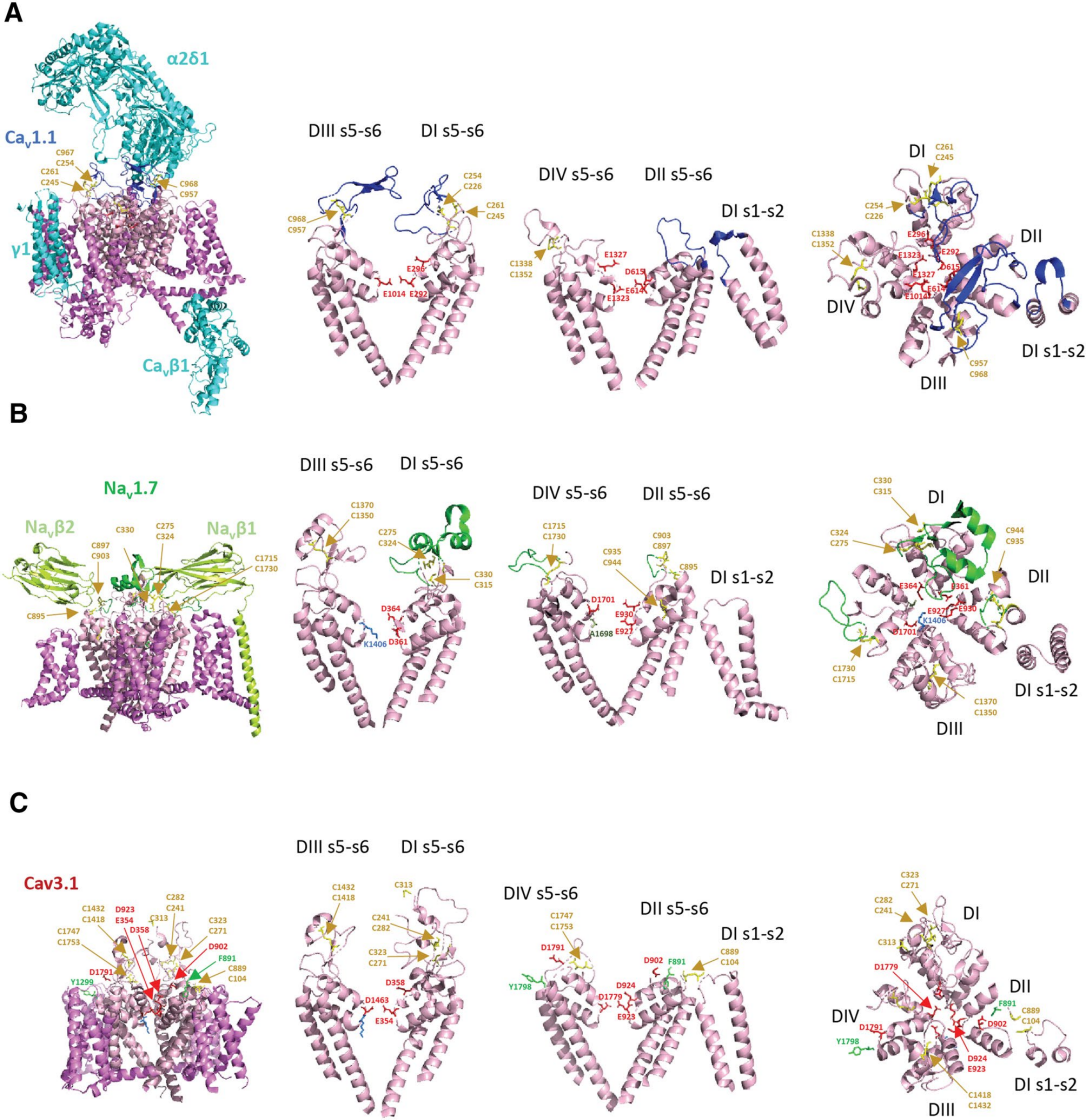
High resolution structures of (**A**) rabbit Ca_v_1.1 channel (PDB: 6jpa, 2.6 Å resolution, (Zhao^20^), (**B**) human Nav1.7 channel (PDB: 6j8g), 3.2 Å resolution (Shen^21^) and (**C**) human Cav3.1 channel (PDB: 6kzp , 3.1 Å resolution (Zhao et al.^18^). PDB files are illustrated using PyMOL Molecular Graphics System, Version 2.3, Schrödinger, LLC, https://pymol.org/2/. Left panels: side view of nanoparticle channel complex with voltage-sensor domains (magenta color) and pore loop domains (light pink color) with bound accessory subunits: Ca_v_β1, Ca_v_α_2_δ_1_(light blue color): Na_v_β1, Na_v_ β2 (light green color). Extracellular loop regions of ion channels bound to accessory subunits are highlighted darker blue (for Ca_v_1.1) and highlighted darker green (for Na_v_1.7). Cysteine bonded pairs in the extracellular loops are labelled (mustard yellow color), with key negatively-charged (red color) and positively-charged (blue color) residues of the pore selectivity filter, indicated. Middle panels (side view) and right panel (top down view) of the opposing DI–DIII and DII–DIV pore loops alone (s5-P-s6), plus DI s1–s2 loop which contains a cysteine bonded to a cysteine in DII L5 (s5-P) loop in Ca_v_3.1. The positioning of the unique cysteine-rich extracellular loops contained in non-vertebrate Ca_v_3 channels in DII s5-P (between F891–D902) and DIV P-s6 (between D1791–Y1798) are labelled, by darker-purple dotted lines.

The overall three-dimensional folding structures of the extracellular loops looming above the conducting ion pores of vertebrate L-type Ca^2+^ channels^20^ (Fig. 11a), (B) human Na_V_1.7 channel^21^ (Fig. 11b) (C) human Ca_V_3.1 channel^18^ (Fig. 11c) differ from each other, but there is a common placement of intra-domain cysteine bridges in extracellular loops in these differing ion channel types of which there are two in D1L5, one in D3L5 and one (likely) in D4L6. The D4L6 cysteine pair so far remains unresolved in the high-resolution structure of Ca_V_3.1 (Supplementary Figure S4). Intra-domain cysteine bridges within extracellular loops are expected to provide structural stability in support of binding the large extracellular domain of the α2 subunit of the Ca_V_α2δ gene, spanning contacts that encompass D1L5, D2L5 and D3L5 extracellular loops of Ca_V_1.1^20^ (Fig. 11a). The extracellular loops of Na_V_1.7 channel are also largely occupied in binding accessory subunits, in this case, Na_V_β subunits, of which the Na_V_β1 and Na_V_β2 subunits span D1L5, D2L5 and D4L6 extracellular loops (Fig. 11b). Ca_V_3.1 channel are not associated with any known accessory subunit, and the configuration of extracellular loops in D1L5 and D3L5 of the Ca_V_3.1 channel are structurally incompatible with binding to the Ca_V_α2 subunit (Supplementary Figure S4)^18^. One would suspect that since T-type channel extracellular loops are not structurally constrained to associate with known accessory subunits, one would expect greater structural flexibility in their extracellular loops. But in fact, the opposite appears to be the case.

Ca_V_3.1 channel notably varies from Na_V_1.7 and Ca_V_1.1 channels, in possessing a cysteine bridge between the extracellular loop of Domain I s1–s2 (C104) in the voltage sensor domain and the singular cysteine of the extracellular loop in D2L5 (C889) of the pore domain (Fig. 11c, Supplementary Figure S4). A striking pattern of cysteine conservation involving Domain I s1–s2 and D2L5 is evident in amino acid alignments of 76 Ca_V_3 channels from different species (Supplementary Figure S5). All cysteines of extracellular loops are lacking in Domain I s1–s2 and D2L5 in basal single cell choanoflagellates and cnidarians (Supplementary Figure S5). The appearance of alternative exon 12a from pseudo-coelomate invertebrates to hemichordates (Supplementary Figure S5), provides what appears to be an additional intra-domain cysteine bridge spanning nine amino acids, between F891 and D902 in the D2L5 extracellular loop of Ca_V_3.1 (Fig. 11c, Supplementary Figure S4). The region between F891 and D902 in Ca_V_3.1 remains unresolved in its published, high resolution structure^18^, indicating that the D2L5 extracellular region is likely a highly flexible structure in vertebrate Ca_V_3.1 channel.

### How does the D2L5 extracellular loop contained between a unique pair of cysteines in alternative exon 12a within the invertebrates generate high Na^+^ current passing Ca_V_3 T-type channels?

The selectivity filter of T-type channels is more constricted than the L-type Ca^2+^ channels with a van der Waals diameter of 2 Å, which forces Ca^2+^ ions to pass through T-type channels as fully dehydrated Ca^2+^ ions^18^. The constricted pore in Ca_V_3 channels is defined by the pore selectivity filter residues in DI (E354) and D3 (D1463), whereas the pore selectivity filter defined by E923 in D2 and D1779 in D4 is twice as wide, approximating 4 Å^18^ (Fig. 11c). It is possible that the optional, extra cysteine bridge pairs in extracellular loops of D2 and D4 constrains the orifice size of the pore selectivity residues of invertebrate Ca_V_3 channels, influencing the relative passage of Ca^2+^ and Na^+^ ions through the pore selectivity filter. Also noteworthy is that the extracellular loops of opposing D2 and D4 domains are dramatically lower in profile above the membrane, and lying in much closer proximity to the pore selectivity filter, compared to the D1 and D3 where the extracellular loops loom much higher above the pore selectivity filter (Fig. 11c). The unique cysteine loop containing 9 amino acids of exon 12a of invertebrate Ca_V_3 channels spans the unresolved D2L5 extracellular loop in Ca_V_3.1 between F891 and D902. Both F891 and D902 are within 1 and 2 amino acids from the negatively-charged aspartate residue (D924) in a key position to attract incoming Ca^2+^ ions. The position of D924 in Ca_V_3.1 channel is universally-featured in all calcium-selective channels, located just above the pore selectivity residue E923 in Ca_V_3.1. We propose that the uniquely structured 9 amino acid cysteine loop in D2L5 of invertebrate Ca_V_3 channels may bring its positive residues (arginine or lysine residues) in proximity to neutralize the aspartate residue (D924) in a manner that dampens the Ca^2+^ current passing capability and promotes a higher Na^+^ current passing capability, as we observed in the D975N Ca_V_3.2 mutant (Fig. 7). We speculate a neutralizing role of the equivalent position of D975 in Ca_v_3.2 channels with positively-charged residues in the D2L5 extracellular loop of invertebrate Ca_V_3–12a channels, although we caution that we did not directly test this possibility within this manuscript.

### Why do all invertebrate T-type channels possessing the major Na^+^ current passing channel engendered with exon 12a always also possess an extra cysteine pair of cysteines in the D4L6 loop compared to vertebrate T-type channels?

We found that exon 12a from invertebrates only generates a high Na^+^ current passing channel in the normally high Ca^2+^ passing Ca_V_3.2 background, when the D4L6 extracellular loop of invertebrate LCa_V_3 is also swapped into vertebrate Ca_V_3.2 channels. The extra pair of cysteines in D4L6 extracellular loop of invertebrate LCa_V_3 channels is located within the unresolved sequence region of Ca_V_3.1 spanning D1791 and Y1798 (Fig. 7a,b). Residues spanning between D1791 and Y1798 are very distant to influence the pore selectivity filter residues directly in Ca_V_3.1, unlike the cysteine-spanning loop within D2L5 of invertebrate LCa_V_3 channels. How the D4L6 extracellular loop working in concert with the D2L5 extracellular loop in opposing domains to influence the relatively high Na^+^ current passing capabilities may be resolved in the cryo-electron microscopy of invertebrate T-type channels.

### The extra cysteine pair (of exon 12a) and two cysteine pairs (of exon 12b) contained in the D2L5 extracellular loops of invertebrate LCa_V_3 channels influence the relative Na^+^ and Ca^2+^ current sizes, and the relative divalent ion current sizes (Ca^2+^, Ba^2+^***Sr***^2+^) and blockade (Ca^2+^, Ni^2+^, Zn^2+^)

We observe that alanine substitution of cysteines in Δcys mutants of the tri-cysteine and penta-cysteine D2L5 loops, spanning exon 12a and exon 12b generates strikingly similar phenotypes, even though these two D2L5 loops contribute to very different phenotypes in native conditions, involving a high Na^+^ current (exon 12a) or more high Ca^2+^ current (exon 12b) passing capacity respectively onto snail LCa_V_3 channels. The Δcys mutants possess a common phenotype include a high permeability of outward monovalent ion currents (Li^+^, Na^+^, K^+^) compared to inward Ca^2+^ currents. With increasing external Ca^2+^ ions to 10 µM, we observed a severely weakened block of the Ca^2+^ ion of the Na^+^ current. Further evidence of altered preferences for ion passage through Δcys mutants is the differing peak sizes of ionic currents with divalent cations as the charge carrier, where Ca^2+^ are larger-sized currents in the Δcys mutants and Ba^2+^ and Sr^2+^ are larger-sized currents in wild type LCa_V_3 channels (Fig. 5).

We observed not only differences in the relative current sizes generated in response to differing external divalent ions (Ca^2+^, Ba^2+^, Sr^2+^) in Δcys mutants but the potency and drug characteristics of the blockade of the Ba^2+^ currents by divalent ions (Ni^2+^ and Zn^2+^) (Fig. 6). Δcys mutants possess an increase in potency of Ni^2+^ and Zn^2+^ blockade. The native snail LCa_V_3 channels possesses a weak blocking capacity for Ni^2+^ and Zn^2+^ resembling mammalian Ca_V_3.1 and Ca_V_3.3 channels, and then increase to the ~ 50 and ~ 10-fold higher Ni^2+^ and Zn^2+^ potency of native mammalian Ca_V_3.2T-type channels^15^, in the Δcys mutants. With higher concentrations of Zn^2+^, we observed a correspondingly greater drug-induced slowing of inactivation kinetics shared between native LCa_V_3, Ca_V_3.1 and Ca_V_3.3 channels. Both the Δcys mutant channels of LCa_V_3 and native Ca_V_3.2 channels possess a higher Ni^2+^ and Zn^2+^ potency, and lacking the slowing of inactivation kinetics associated with Zn^2+^ blockade^16^.

### Zn^2+^ and Ni^2+^ blockade, the redox sensitivity of Ca_V_3.2 and consequences of cysteine mutations of loop cysteines in LCa_V_3 may to be linked

Our discovery of a similar potency and characteristics of the blockade with divalent cations (Ni^2+^, Zn^2+^) between LCa_V_3–Δcys and Ca_V_3.2 channels is relevant in context of the published findings of the unique sensitivity of Ca_V_3.2 channels amongst mammalian Ca_V_3 channels to oxidation of its disulphide bonds within extracellular loops*.* Oxidizing agents like s-nitrosothiols or lipoic acid or dithionitrobenzoic acid (DTNB) reduce the size of T-type channel currents when applied, and this inhibition of currents can be relieved by mutation of unique, extracellular cysteines contributed from the Domain I s1–s2 linker^15,22^, the D1L5 loop^23^, and the D2L5 loop^15,24^. Thiol group reduction with dithiothreitol (DTT) or l-cysteine has the opposite effect, enhancing T-type channel currents, by a means that is specific to Ca_V_3.2 channels, and is prevented by mutations that alter the high affinity Zn^2+^and Ni^2+^ block of Ca_V_3.2 channels, at a particular histidine residue (H191) within an Asp–Gly–His motif in the Domain I, s3–s4 extracellular loop of mammalian Ca_V_3.2^24^. Both cysteine mutations of the extracellular D2L5 in snail LCa_V_3 and the H191 mutation in Domain I, S3–S4 loops of Ca_V_3.2 appear to be generating a strikingly similar phenotype. Substitution of D2L5 extracellular loop cysteines, increases Zn^2+^and Ni^2+^ binding affinity of snail LCa_V_3 channels, whereas, mutation of H191 in the D1, S3–S3 extracellular loop of Ca_V_3.2 decreases Zn^2+^and Ni^2+^ binding affinity with consequences of a lowered sensitivity of their disulphide bonds in its cysteine-enriched extracellular loops to oxidation.

### A possible evolution of the configuration of unique cysteine bridges amongst different Ca_V_3 channels

The simplest organisms with a Ca_V_3 channel are the single-cell choanoflagellates, and they lack two sets of cysteine pairs in their extracellular loop. Choanoflagellate Ca_V_3 channels are lacking one universal cysteine pair in the DIL5 extracellular loop found in other Ca_V_3 channels (**Supplementary Figure 4), and lacking the unique cysteine bridge pair between the voltage sensor domain (C104, D1s1–s2) and the pore domain (C889, D2L5), present in Ca_V_3.1 and other vertebrate Ca_V_3 channels (Supplementary Figures S1, S2, S5). The unique C104–C889 bridge pair between the voltage-sensor and pore domain is also lacking amongst the cnidarians. Instead, cnidarians possess two different Ca_V_3 genes, unlike most other invertebrates which contain only one Ca_V_3 gene in their genome. One cnidarian gene isoform resembles the vertebrate conditions with one cysteine pair in the D4L6 extracellular loop and one gene isoform resembling the condition within all other non-vertebrates, including the single-cell choanoflagellates with two cysteine pairs located in the D4L6 extracellular loop (Supplementary Figures S1, S4, S6). The protostome invertebrates, including the basal Platyhelminthes (eg. free living flatworm, *Macrostomum lignano*) to the basal chordates (eg. tunicate, *Ciona intestinalis*) contain one or both of alternative exon 12a and exon 12b (Supplementary Figures S1, S5), after an intron splice junction separating exons 11 and 12 appeared in common ancestors of the *Platyhelminthes*. Alternative exons 12a provides the equivalent D2L5 extracellular cysteine (C889) that bridges to C104 of the voltage-sensor domain found in Ca_V_3.1 channels (C209–C0151 in LCa_V_3–12a, Supplementary Figure S4, red color), and also provide its own 9 amino acid sequence flanked by cysteines spanning F891 and D902 in D2L5 (C1054–1064 in LCa_V_3–12a, Supplementary Figure S4, brown color).

We found that the alternative exon 12a increases the Na^+^ current size through Ca_V_3 channels. All invertebrate Ca_V_3 channels lacking the equivalent D2L5 extracellular cysteine (C889), such as those containing exon 12b possess what appears to be an alternative cysteine location in D2L5 extracellular loop (see red colored residues, Supplementary Figure S5), that is expected to bridge to equivalent to C104 of the voltage-sensor domain found in Ca_V_3.1 channels (C209–C1062 in LCa_V_3–12b, Supplementary Figure S4, red color). These Ca_V_3 channels with exon 12b also contain the equivalent D2L5 loop flanked by cysteines (C1054–C1075, Supplementary Figure S4, brown color) found in exon 12a, but also always possess an additional cysteine pair (C1064–C1073 in LCa_V_3–12b, Supplementary Figure S4, blue color). We found through swaps of D2L5 sequences in the Ca_V_3.2 and LCa_V_3–12a, and LCa_V_3–12b background that exon 12b, containing its three proposed cysteine pairs, including (C209–C1061) which appear to be equivalent to the single cysteine pair (C104–C889) of Ca_V_3.1 and two cysteine pairs confined to D2L5 (C1054–C1075, and C1064–C1073), promotes a relative Ca^2+^ current size approaching that of human Ca_V_3.2 channels. In comparison to LCa_V_3–12b, vertebrate Ca_V_3.2 channels possess a limited configuration of a single cysteine pair (C104–C889) spanning D1s1–S2 and D2L5 in human Ca_V_.3.1 channels, and one cysteine pair in D4L6. The two proposed cysteine pairs in exon 12a are C209–C1051 (equivalent to C104–C889 in Ca_V_3.1), and C1054–C1064 confined to D2L5, causes an increase in relative Na^+^ current size through Ca_V_3 channels. We found the highest relative sized Na^+^ current in the presence of the extra cysteine pair in D4L6 extracellular loop (C2056–C2060, Supplementary Figure S4, purple color), which is an extra cysteine pair found in all non-vertebrates. Some non-vertebrate species contain a variation that includes some but not all the cysteine pairs in the extracellular loops (eg. placozoan, *Trichoplax adherens*, nematodes Ca_V_3–12b isoforms: *Strongyloides stercoralis* and *Caenorhabditis elegans* or the cephalochordate *Branchiostoma lanceolatum*) (Supplementary Figure S5).

### Novel cysteine bridge pairs in extracellular loop spanning D1s1–s2, D2L5 and D4L6 in Ca_V_3 channels are structurally independent

We demonstrate from our D2L5 and D4L6 loop swaps on mammalian Ca_V_3.2 and invertebrate LCa_V_3 channels that the extracellular loop pairs, operate in a complementary and additive manner, contributing to a change in the relative passage of Ca^2+^ or Na^+^, depending on whether the extracellular loop was derived from a highly Ca^2+^ current passing or high Na^+^ current passing Ca_V_3 T-type channel. If there was an inter-dependence of cysteines amongst domains, we may have expected that the xeno-grafting of individual or dual extracellular loops would uniformly disable the proper folding of extracellular loops and the functional expression of Ca_V_3 channels. Our observed functional independence of D2L5 and D4L6 loops is consistent with the likely independent evolution of D2L5 loops within common ancestors of non-cnidarian invertebrates, and D4L6 loops in cnidarians, without consequences to the integrity of the Ca_V_3 channel structure as a whole.

The extra cysteine pairs in extracellular loops of Ca_V_3 channels, compared to other Ca^2+^ channels (Ca_V_1 and Ca_V_2) and to Na^+^ channels (Na_V_2 and Na_V_1) is a likely reflection of the constraints on the flexibility of extracellular loops looming above the pore domain of Ca_V_3 channels, in spite of the absence of required auxiliary subunit binding that occupies most of the extracellular loop domains of Na^+^ (Na_V_1) and Ca^2+^ (Ca_V_1 and Ca_V_2) channels. Notably, the enrichment of cysteines in extracellular loop of invertebrate Ca_V_3 channels are restricted to D2L5 and D4L6 extracellular loops, in opposing domains, D2 and D4, which have a closer proximity to the pore selectivity filter than the other opposing domains, D1 and D3 (Fig. 11c).

As we have observed here, the dramatic enrichment and diversity of cysteine pairs in extracellular loops looming above the pore of invertebrate Ca_V_3 channel contribute to a dramatic change in the contribution of the relative current sizes of monovalent ion currents (Li^+^, Na^+^, K^+^, Cs^+^) and divalent ion (Ca^2+^, Ba^2+^, Sr^2+^) currents through Ca_V_3 channel pores, and also changes the potency and characteristic of the block by divalent ions (Ca^2+^, Ni^2+^, Zn^2+^). The large number of cysteine pairs in extracellular domains likely contributes to an observed unique regulation of Ca_V_3 channel activity by oxidation of its disulphide bonds. Future high-resolution structures will clarify the unique structural diversity of cysteine bridges in extracellular loops of non-mammalian Ca_V_3 channels. Further exploration will help to explain fully why Ca_V_3 channels, especially in invertebrates, invested in such a large and diverse set of cysteine pairs in extracellular loops, given that they are not necessary in Ca_V_3 channels for accommodating large auxiliary subunits of the Na^+^ (Na_V_1) and Ca^2+^ (Ca_V_1 and Ca_V_2) channels.

## Methods

### Cloning and expression of snail LCa_V_3 channels

The original, mostly Ca^2+^ current passing isoform of the invertebrate LCa_V_3 T-type channel (GenBank Accession #: AF484084), isolated from pond snail, Lymnaea *stagnalis*, was expressed and characterized in a configuration contained exon 12b, as well as optional exon 18b spanning the I–II linker, but lacking exon 25c of the III–IV linker^7^. LCa_V_3 T-Type Ca^2+^ channels containing exon 12b, but lacking in exon 8b (GenBank Accession # JQ313138) and containing exon 25c (GenBank Accession # JQ313139) were subsequently described in Senatore and Spafford^8^. Novel exon 12a isoform (+ 8b, − 25C) deposited as GenBank Accession # JX292155, is compared with exon 12b isoform (+ 8b , − 25C) which is the configuration of the three exons that is more commonly expressed in the snail brain than in the snail heart, where there is exclusive expression of the mostly Na^+^ current passing Ca_V_3 T-type channel with exon 12a^9^. mRNA isolation and quantitation of mRNA expression from juvenile and adult snail tissue has been described previously^9^. Chimeras channels were generated by swapping synthetic gene fragments (ordered from BioBasic) at uniquely engineered restriction enzyme sites, as explained below:

### Construction of D2L5 loops lacking cysteine residues in snail LCa_V_3 channels

LCa_V_3–12b (9031 bp mRNA transcript) was subcloned into pGEMT vector with unique BglII and SalI restriction sites (positions: 1,391–4,521) and novel silent restriction sites AvrII and Eco47III (AfeI) were created by Quikchange mutagenesis (Stratagene, Agilent Technologies) that cut at (positions: 3,338–3,567) spanning the coding sequence for exon 12a (39 aa) and exon 12b (50 aa). Synthetic DNA (ordered from BioBasic International) spanning the AvrII and Eco47III restriction sites were inserted into the LCa_V_3 subclone, which included D2L5 loop changes for LCa_V_3–12a Δcys, LCa_V_3–12b Δcys each of which contained a silent NruI and MluI restriction sites, respectively, for rapid validation of individual cloned plasmid stocks for their unique D2L5 loop identity in LCa_V_3.

### Creation of D975N mutant in human Ca_V_3.2 channels

Human Ca_V_3.2, coded by CACNA1H gene (7,762 bp mRNA transcript) of Genbank Accession # AF051946, was subcloned (from positions: 342–3,921) into pBluescript II vector from restriction sites spanning NotI and AgeI restriction sites in the Bluescript vector polylinker generated by synthetic oligo insertion. Novel silent restriction sites AvrII was created into human Ca_V_3.2 subclone by Quikchange mutagenesis (Stratagene, Agilent Technologies), and combined with native BsrGI restriction site downstream of the AvrII site, cut out the region (positions: 2,873–3,018) spanning the homologous exon 12a (39 aa) and exon 12b (50 aa) in snail LCa_V_3 channels. Synthetic DNA (ordered from BioBasic International) spanning the AvrII and BsrGI restriction sites were inserted into the human Ca_V_3.2 subclone, which included a D975N substitution, contained a silent AgeI restriction site for rapid validation of individual cloned plasmid stocks for their unique D2L5 loop identity in human Ca_V_3.2.

### D2L5 mutagenesis in snail LCa_V_3–12a/12b backgrounds

LCa_V_3–12b (9031 bp mRNA transcript) was subcloned into pGEMT vector with unique BglII and SalI restriction sites (positions: 1,391–4,521) and novel silent restriction sites AvrII and Eco47III (AfeI) were created by Quikchange mutagenesis (Stratagene, Agilent Technologies) that cut at (positions: 3,338–3,567) spanning the coding sequence for exon 12a (39 amino acids) and exon 12b (50 amino acids). Synthetic DNA (ordered from BioBasic International) spanning the AvrII and Eco47III restriction sites were inserted into the LCa_V_3 subclone, which included D2L5 loop changes for human Ca_V_3.2 Genbank Accession # AF051946 (positions: 2,873–3,069), each of which contained a silent NruI, MluI, AgeI restriction sites, respectively, for rapid validation of individual cloned plasmid stocks for their unique D2L5 loop identity in LCa_V_3.

### D2L5 mutagenesis in human Ca_V_3.2 backgrounds

Human Ca_V_3.2, coded by CACNA1H gene (7,762 bp mRNA transcript) of Genbank Accession # AF051946, was subcloned (from positions: 342–3,921) into pBluescript II vector from restriction sites spanning NotI and AgeI restriction sites in the Bluescript vector polylinker generated by synthetic oligo insertion. Novel silent restriction sites AvrII was created into human Ca_V_3.2 subclone by Quikchange mutagenesis (Stratagene, Agilent Technologies), and combined with native BsrGI restriction site downstream of the AvrII site, cut out the region (positions: 2,873–3,018) spanning the homologous exon 12a (39 amino acids) and exon 12b (50 amino acids) in snail LCa_V_3 channels. Synthetic DNA (ordered from BioBasic International) spanning the AvrII and BsrGI restriction sites were inserted into the human Ca_V_3.2 subclone, which included D2L5 loop swaps for snail LCa_V_3 exon 12a and LCa_V_3 exon 12b and a D975N substitution, each of which contained a silent DpnI, MluI and AgeI restriction sites, respectively, for rapid validation of individual cloned plasmid stocks for their unique D2L5 loop identity in human Ca_V_3.2.

### D4L6 mutagenesis in snail LCa_V_3–12a/12b backgrounds

Snail LCa_V_3–12b was subcloned into pGEMT vector (at positions: 4,522–6,858) with unique SalI restriction site and a silent MluI restriction site created in the original concatenation of cDNA fragments to generate full length LCa_V_3–12b. Novel silent restriction sites BbsI and XhoI were created by Quikchange mutagenesis (Stratagene, Agilent Technologies) that cut at (positions: 3,338–3,567) spanning the coding sequence for the LCa_V_3 D4L6 loop. Synthetic DNA (ordered from Biobasic International) spanning the BbsI and XhoI restriction sites were inserted into the LCa_V_3 subclone, which included D4L6 loop changes coding for 16 amino acids (positions: 5,540–5,587) for human Ca_V_3.2 to generate LCa_V_3 D4L6H which contained a silent MfeI restriction sites, for rapid validation of individual cloned plasmid stocks for the unique identity of LCa_V_3 D4L6H.

### D4L6 mutagenesis in human Ca_V_3.2 backgrounds

The complete C terminus of human Ca_V_3.2 (at positions: 3,922–7,138) was subcloned into pBluescript vector in a modified polylinker utilizing a unique AgeI restriction site in the coding region and a downstream XbaI site only present in the pcDNA 3.1 mammalian expression vector. Silent restriction sites for XhoI was created in the Ca_V_3.2 subclone by synthetic DNA insertion of a SphI and AvrII cDNA fragment (ordered from BioBasic International) into the Ca_V_3.2 subclone that allowed for insertion of another DNA synthetic construct (ordered from Biobasic International) between SphI and XhoI (positions: 5,285–5,666 in Ca_V_3.2) spanning the 20 amino acids coding sequence for the LCa_V_3 D4L6 loop (positions: 6,386–6,442 in LCa_V_3). Ca_V_3.2 D4L6L contained silent DraII and DpnI restriction sites, for rapid validation of individual cloned plasmid stocks for the unique identity of LCa_V_3 D4L6H.

### Phylogenetic analyses

Ca_V_3 channel genes were derived from transcriptome or genome sequences available by BLAST searching of publicly available websites: GenBank (NIH genetic sequence database), DOE JGI (U.S. Department of Energy Joint Genome Institute) and EnsembleGenomes (EBI and Wellcome Trust Sanger Institute). Multiple alignments were carried out in using MUltiple Sequence Comparison by Log-Expectation (MUSCLE) at website: https://www.ebi.ac.uk/Tools/msa/muscle/^25^. and gene trees were created with Phylogeny.fr at website: https://www.phylogeny.fr/^26^.

### Statistical analyses

Numerical values in electrophysiological experiments were compared using a parametric one-way ANOVA within OriginPro 2018 (64-bit) SR1 b9.5.1.195. (OriginLabs). Statistical significance by means comparison was conducted using a Tukey post hoc test, unless otherwise stated.

### PDB file illustrations

PDB files are illustrated using PyMOL Molecular Graphics System, Version 2.3, Schrödinger, LLC, https://pymol.org/2/.

### In vitro expression of Cav3 channels in HEK-293T cells

We have detailed our optimized technique for the culture and maintenance of mammalian HEK-293T cells, Ca^2+^ phosphate transfection and expression of ion channels, and their recording using whole-cell patch clamp in an online video journal^27^.

Snail LCa_V_3 clones including chimeric and mutated derivatives were expressed within bi-cistronic pIRES2-EGFP vector, which generates green fluorescence upon mercury lamp excitation of positively transfected cells. Human Ca_V_3.1, Ca_V_3.2 and Ca_V_3.3 channels contained in pcDNA3.1 vector, were co-expressed with a pTracer plasmid, which provided green fluorescence for identification of positively transfected cells. Expressed human Ca_V_3 channels were originally a gift from Dr. Edward Perez-Reyes (U. of Virginia, Charlottesville) and Dr. Gerald Zamponi (U. of Calgary, Calgary).

All transient transfection procedures, external and internal solutions, voltage-protocols for electrophysiology, post-recording and phylogenetic analyses were carried out as stated previously, including the primary cell culture of snail heart cells^9^. Ca^2+^ channels were expressed in human embryonic kidney (HEK293T) cells via Ca^2+^ phosphate transfections.

### Whole cell patch clamp recordings

Whole cell electrophysiology recordings were obtained using an Axopatch 200B or Multiclamp 700B amplifiers (Molecular Devices), sampled through a Digidata 1440a A/D converter (Molecular Devices) to a PC computer. Patch pipettes for recording had pipette resistances of 2–5 MΩ (HEK-293 T cells) or 5–10 MΩ (heart cells), and with typical access resistance maintained after breakthrough between 2.5 MΩ and 6 MΩ (HEK-293 T cells) or 10 and 14 MΩ (heart cells). Only recordings with minimal leak (< 10% of peak) and small current sizes (< 500 pA) in HEK-293 T cells were used due to loss of voltage clamp above 500 pA. Series resistance was compensated to 70% (prediction and correction; 10-µs time lag). Offline leak subtraction was carried out using the Clampfit 10.2 software (Molecular Devices). For all recordings, offline leak subtraction was carried out and data was filtered using a 500 Hz Gaussian filter in Clampfit 10.2. Reversal potentials were calculated in bi-ionic conditions using a calculation of relative permeabilities, as described previously^9^. Protocols for measuring the voltage-sensitivity and kinetics, and curve fitting data are described previously^9^ A Valvelink 8.2 gravity flow Telfon perfusion system (AutoMate Scientific) was utilized to compare ionic current sizes generated by differing external monovalent or divalent ions.

### Ionic solutions used in electrophysiology experiments

Divalent cations for Ca_V_3 channel blockade were purchased from Sigma-Aldrich: Nickel (II) chloride hexahydrate (Cat #: 203,866) and zinc chloride (Cat #: 229,997). The standard recording solutions to measure ionic conductances with external calcium or barium or strontium ions (in Figs. 1, 5, 6, 7) is shown in Table 1.

**Table 1:** Recording solutions to measure calcium or barium conductances (Figs. 1, 5, 6, 7).

CaCI_2_	BaCI_2_	SrCI_2_	TEA-CI	HEPES		
**External**^a^						
2	0	0	160	10		
0	2	0	160	10		
0	0	2	160	10		

#	CsCI	NMDG+	EGTA	Mg-ATP	Li-GTP	HEPES
**Internals**^b^						
1	110	0	10	3	0.6	10

^a^pH 7.4 with TEA-OH.

^b^pH 7.2 with CsOH.

The relative inward monovalent ion conductance involving sodium compared to calcium ions using NMDG^+^ (measured in Figs. 2, 7, 8) is shown in Table 2.

**Table 2:** Recording solutions to measure relative inward sodium and/or calcium conductances using NMDG+ (Figs. 2, 7, 8).

CaCI_2_	BaCI_2_	NaCI	NMDG+	TEA	HEPES	
**External**^a^						
0	2	0	135	25	10	
0	2	135	0	25	10	
2	0	0	135	25	10	
2	0	135	0	25	10	

#	CsCI	NMDG +	EGTA^b^	Mg-ATP	Li-GTP	HEPES
**Internals**^b^						
1	110	0	10	3	0.6	10

^a^pH 7.4 with TEA-OH.

^b^pH 7.2 with CsOH.

The relative permeability of PCa/Px, where x is monovalent ion (Li^+^, Na^+^, K^+^, Cs^+^) was calculated by the following bi-ionic equation provided on the bottom of page 34 of Fatt and Ginsborg (1958)^10^:

$$\frac{{P}_{Ca}}{{P}_{x}}=\frac{{\left[x\right]}_{i}}{4{\left[Ca\right]}_{o}}exp\left({E}_{rev}F/RT\right)\left\{exp\left({E}_{rev}F/RT\right)+1\right\}$$

The bio Bi-ionic solutions for determining the relative PCa / Px permeabilities (measured in Figs. 3, 9) is shown in Table 3.

**Table 3:** Bi-ionic solutions for determination of relative PCa / Px permeabilities (Figs. 3, 9).

CaCI_2_	TEA-CI	HEPES					
**External**^a^							
4	155	10					

#	CsCI	NaCI	KCI	LiCI	EGTA^b^	TEA-CI	HEPES
**Internals**^c^							
1	100	0	0	0	10	10	10
2	0	100	0	0	10	10	10
3	0	0	100	0	10	10	10
4	0	0	0	100	10	10	10

^a^pH 7.4 with TEA-OH.

^b^pH 7.2 with XOH where X = Cs, Na ,K, or Li.

^c^pH 8.0 with XOH.

The ionic solutions used in Fig. 4 and Fig. 10 to measure the relative change in peak current sizes in response to differing external calcium ion concentrations in the presence of 60 mM external sodium ion is provided in Table 4.

**Table 4:** [Ca ^2+^1ex dose response with 60 mM [Na+]ex (Figs. 4, 10; Supplementary Figures S31, S7).

In mM	Ca^2+^ free	CaCI_2_	NaCI	EGTA	HEPES	Glucose
**External**^a^						
1	1 × 10^−9^	0.01	60	1.246	10	26.3
2	1 × 10^−7^	1.00	60	2.236	10	20.4
3	1 × 10^−5^	1.00	60	1.002	10	24.1
4	1 × 10^−4^	0.10	60	0	10	29.7
5	3 × 10^−4^	0.30	60	0	10	29.1
6	1 × 10^−3^	1.00	60	0	10	27.0
7	3 × 10^−3^	3.00	60	0	10	21.0
8	1 × 10^−2^	10.00	60	0	10	0.0

CsCI	EGTA	Mg-ATP	Li-GTP	HEPES		
**Internals**^b^						
110	10	3	0.6	10		

^a^pH 7.4 with TEA-OH.

^b^pH 7.4 with CsOH.

### Contribution to the field

Variable, and asymmetrical extracellular loops in Ca_V_3 channels possess strategically-positioned charged amino acid residues and intra-loop disulphides, contributing to structures that regulate the relative passage and block of Na^+^ and Ca^2+^ currents.

The weak conservation amongst extracellular loop sequences overall, even amongst closely related homologs of cation channel class of Na_V_, Ca_V_ and NALCN channels, suggest that each eukaryotic cation channel gene has a composition of extracellular loops from different domains that may operate quite uniquely and independently from each other, possibly contributing to different properties such as ion passage, cation drug block and sensitivity to venom toxins. A changeable external scaffold provides for potential adaptation to changing cellular environments within different organisms, without changing the fundamental high field strength (HFS) site of the pore’s selectivity filter, which is a highly conserved and characteristic sequence within different classes of voltage-gated Na^+^ (Na_V_) and Ca^2+^ (Ca_V_) channels, and NALCN. Determination of the functional consequences to the structural differences in nature’s alternative extracellular loops will aid in design of human medicines, and in pest control (yeast, protest, algal and invertebrate) involving the variable external scaffolds of voltage-gated Na_V_, Ca_V_ and NALCN channels as targets.

## Supplementary information

Supplementary information.

## Abbreviations

HSF site: High field strength site
D2L5: Extracellular loop spanning the end of transmembrane segment 5 to the start of the pore selectivity filter (S5-P) in Domain II
D4L6: Extracellular loop spanning the end of the pore selectivity filter to the start of transmembrane segment 6 (P-S6) in Domain IV
subscripts “L” and “H”: Ca_V_3 T-type channel loops in chimeric channels from ***Lymnaea stagnalis*** and **H**uman Ca_V_3.2 channel isoform, respectively, such as D2L5H, D4L6H or D4L6L
LCaV3–12a and LCaV3–12b: The alternative “a” and “b” splice isoforms spanning exon 12, which includes the the distal ends of Domain II, segment 5, the extracellular D2L5 loop, and the proximal, P1 helix before the selectivity filter in Domain II of Lymnaea CaV3 T-type channel
