# Supplementary material for: Unique cysteine-enriched, D2L5 and D4L6 extracellular loops in CaV3 T-type channels alter the passage and block of monovalent and divalent ions
Source: Sci Rep. 2020 Jul 24;10:12404. doi: 10.1038/s41598-020-69197-3 (PMC7382465; doi:10.1038/s41598-020-69197-3)
Supplement: Supplementary file 1 — Supplementary information. [file 41598_2020_69197_MOESM1_ESM.pdf]

Supplementary Figures

Title: “*Unique cysteine-enriched, D2L5 and D4L6 extracellular loops in Ca_V3 T-type channels alter the passage and block of monovalent and divalent ions*”

Authors: Wendy Guan*, Robert F. Stephens*, Omar Mourad, Amrit Mehta, Julia Fux and J. David Spafford#

From the Department of Biology, University of Waterloo, Waterloo, Canada. N2L 3G1

*These two authors contributed equally to the manuscript

#To whom correspondence should be addressed:

J David Spafford,
B1-173, Department of Biology,
University of Waterloo, Waterloo, Ontario,
Canada. N2L 3G1
Tel: 519-888-4567 x 38186;
Fax: 519-746-0614;
E-mail: spafford@uwaterloo.ca

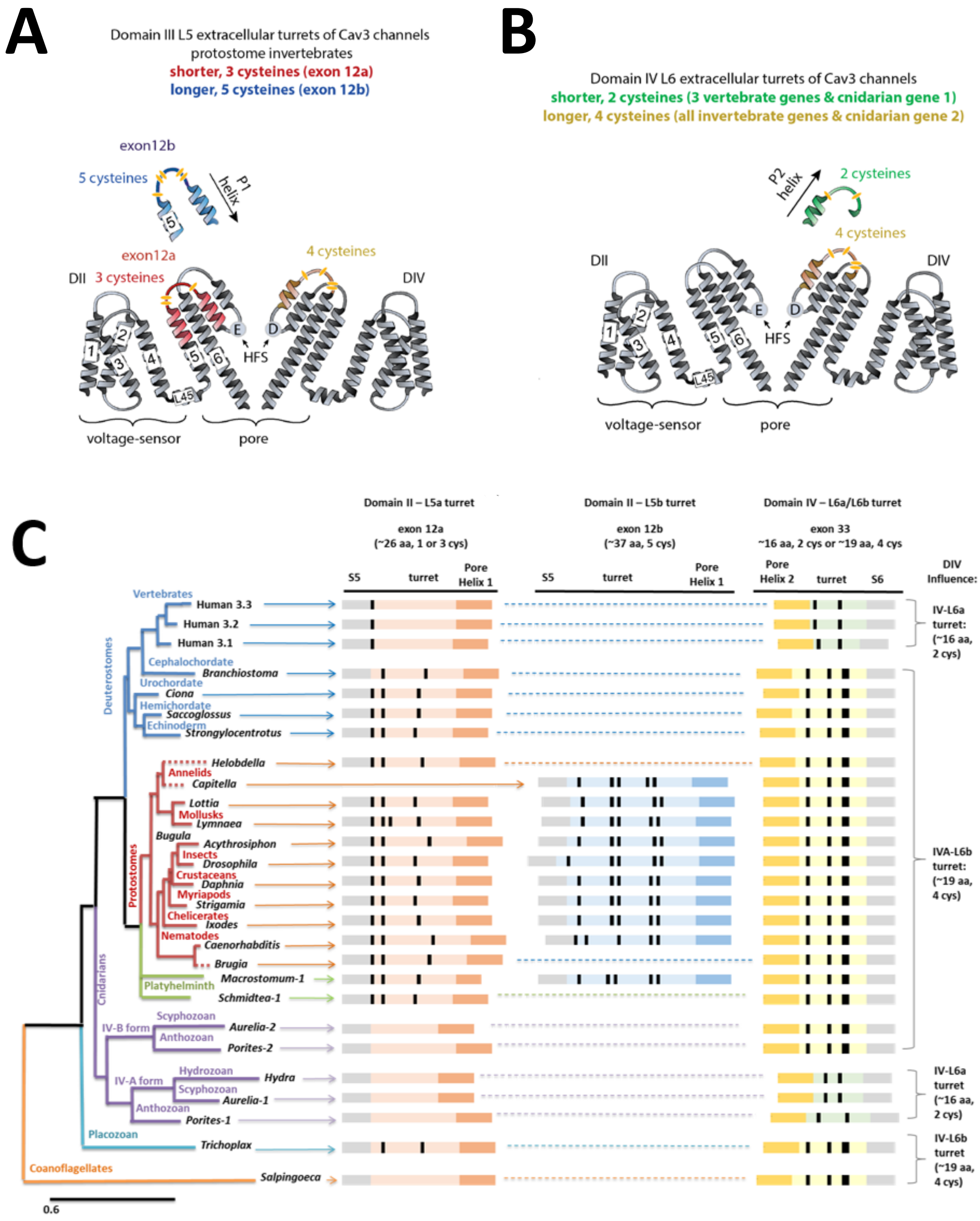

Supplementary Figure 1. Phylogenetic distribution and sequence diversity of alternative extracellular D2L5 and D4L6 extracellular loops in Ca_v3 T-type channels. (A, B) Cartoon illustrating a cut-away, side view of Domain II and IV of Ca_v3 T-type channels with alternative D2L5 extracellular loops coded in exon 12a and exon 12b in protostome invertebrates (A) and alternative D4L6 extracellular loop configurations found in anthozoan and scyphozoan cnidarian gene isoforms (B). The extracellular loops loom above the re-entrant pore containing a critical high field strength (HFS) site which serves as a key regulator of sodium and calcium ion preferences through Ca_v and Na_v channels. (B) Gene tree illustrating extracellular loop sizes with vertical black lines indicating location of cysteine residues. Alternative gene splicing of D2L5 extracellular loops in protostome invertebrates are included in shorter exon 12a (orange color) which are mostly tri-cysteine extracellular loops and a longer exon 12b, (blue color) which are mostly penta-cysteine extracellular loops. D4L6 extracellular loops are di-cysteine in vertebrates (green shaded colors) or tetra-cysteine (yellow shaded colors) in non-vertebrates. Anthozoan and scyphozoan cnidarians possess both the di-cysteine isoform found in vertebrate Ca_v3 channels and the tetra-cysteine isoform of non-vertebrates Ca_v3 channels. Gene tree was generated with Phylogeny.fr (http://www.phylogeny.fr/) [26].

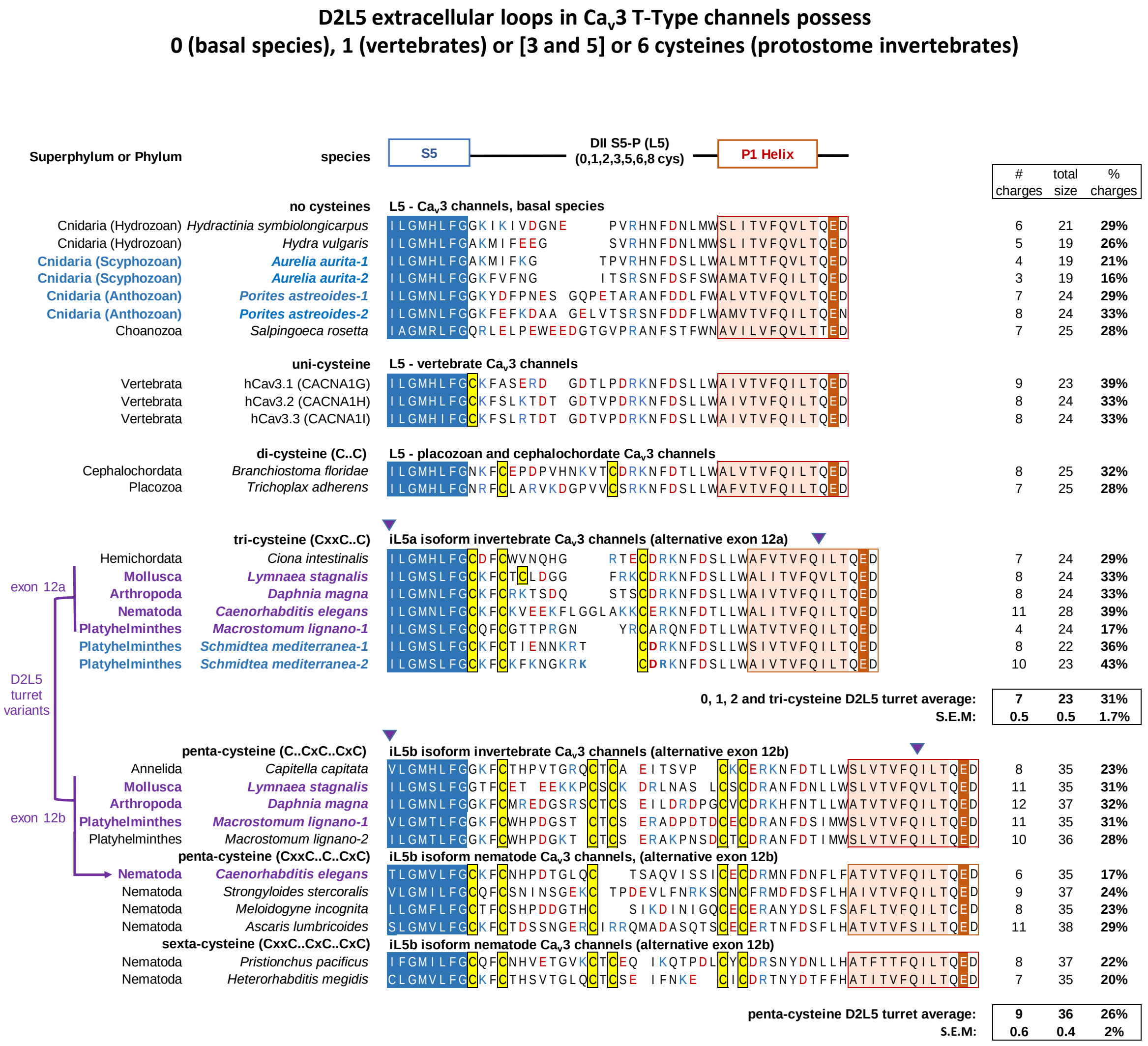

Supplementary Figure 2. Alignment of D2L5 extracellular loops in Ca$_v$3 T-type channels. Extracellular loops sequences between transmembrane segment 5 (**blue**) and the P1 (Pore1) helix (**light orange**) before the selectivity filter (**dark orange**) in Domain II of Ca$_v$3 T-type channels. Pattern of cysteines (**yellow residues**) can be zero (basal species: single cell coanoflagellates, cndiarians), one (vertebrates), two (placozoan and cephalochordates) or alternatively three and five (non-cnidarian, invertebrates), six (some nematodes). The indicated species containing the same or alternative (exon 12a and exon 12b) extracellular loops are outlined in **light blue colored names** or **purple colored names**, respectively. Note the high density of charged amino acid residues (**red and blue color residues**) in D2L5 extracellular loops, which would play a role in attracting and binding of ions within the external scaffold above the re-entrant pore. ~1/4 to 1/3 of all residues in the D2L5 extracellular loop are positively or negatively charged amino acid residues as indicated. The multiple alignments in the figure were created using MUltiple Sequence Comparison by Log- Expectation (MUSCLE) at website: https://www.ebi.ac.uk/Tools/msa/muscle/ [25].

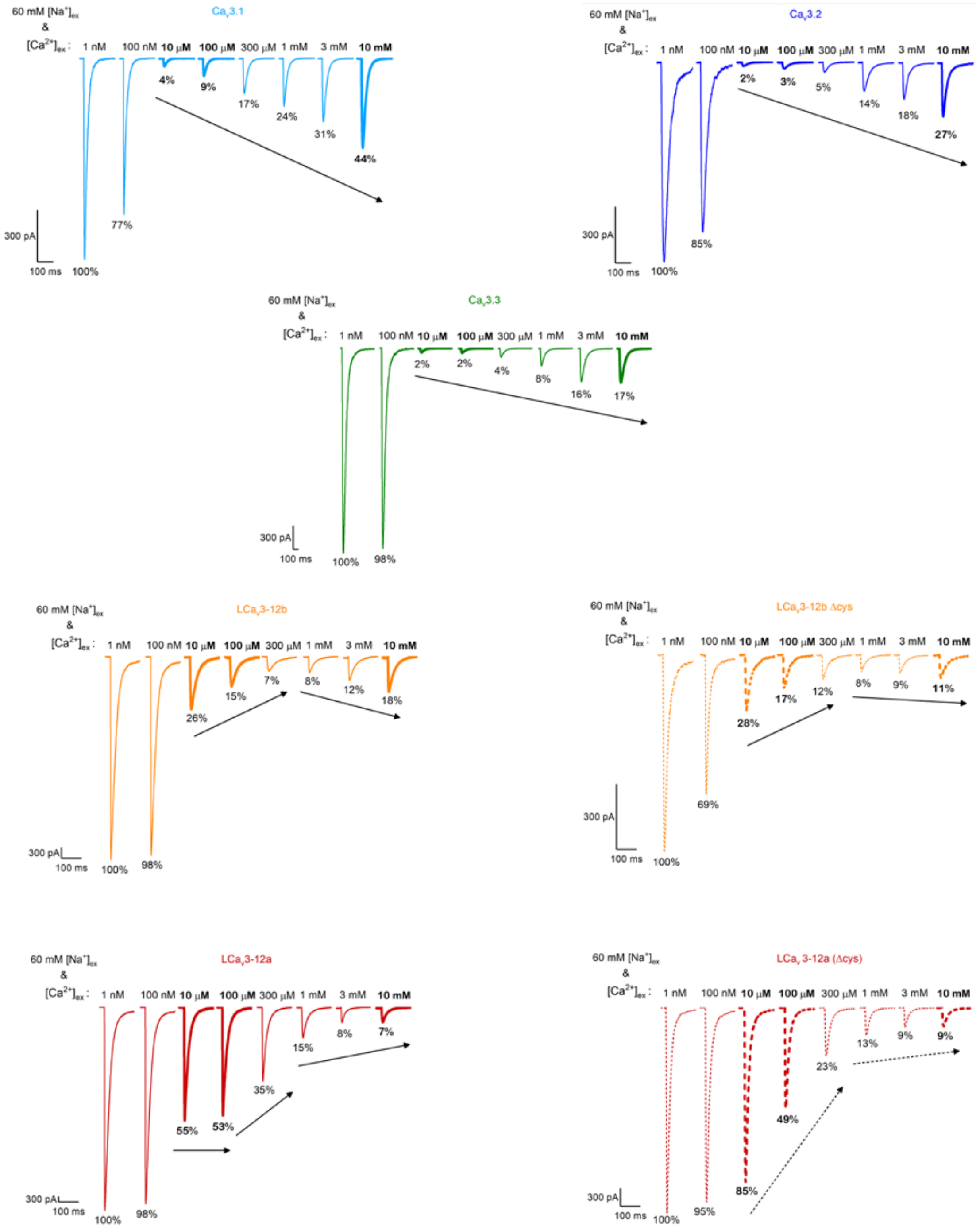

Supplementary Figure 3. Peak current sizes of representative traces of Ca_v3 T-type channel currents in response to the rise in external Ca^{2+} concentrations from 1 nM to 10 mM in the presence of 60 mM external Na^+. Data replicates are collated into graphs in Fig. 6. Human $Ca_v3.1$, $Ca_v3.2$ and $Ca_v3.3$ channel currents dramatically decline in response to 10 µM external Ca^{2+} which is the external Ca^{2+} concentration which almost completely blocks the current carried by external Na^+. From 10 µM and 10 mM external Ca^{2+} peak currents rise as these are Ca^{2+} selective Ca_v3 T-type channels. The snail Ca_v3 T-type channels possess a weaker block of the Na^+ current at 10 µM external Ca2, and possess a continued decline in peak currents in the presence of rising external Ca^{2+} to mM concentrations, reflecting the higher Na^+ passing character of these channels. The current tracings at 10 µM, 100 µM and 10 mM concentrations are highlighted because they reflect the key external Ca^{2+} concentrations that indicate the degree of Ca^{2+} block of the Na^+ current, and relative Ca^{2+} permeability in the physiological (mM range). Data in this figure were analyzed and illustrated using OriginPro 2018 (64-bit) SR1 b9.5.1.195.

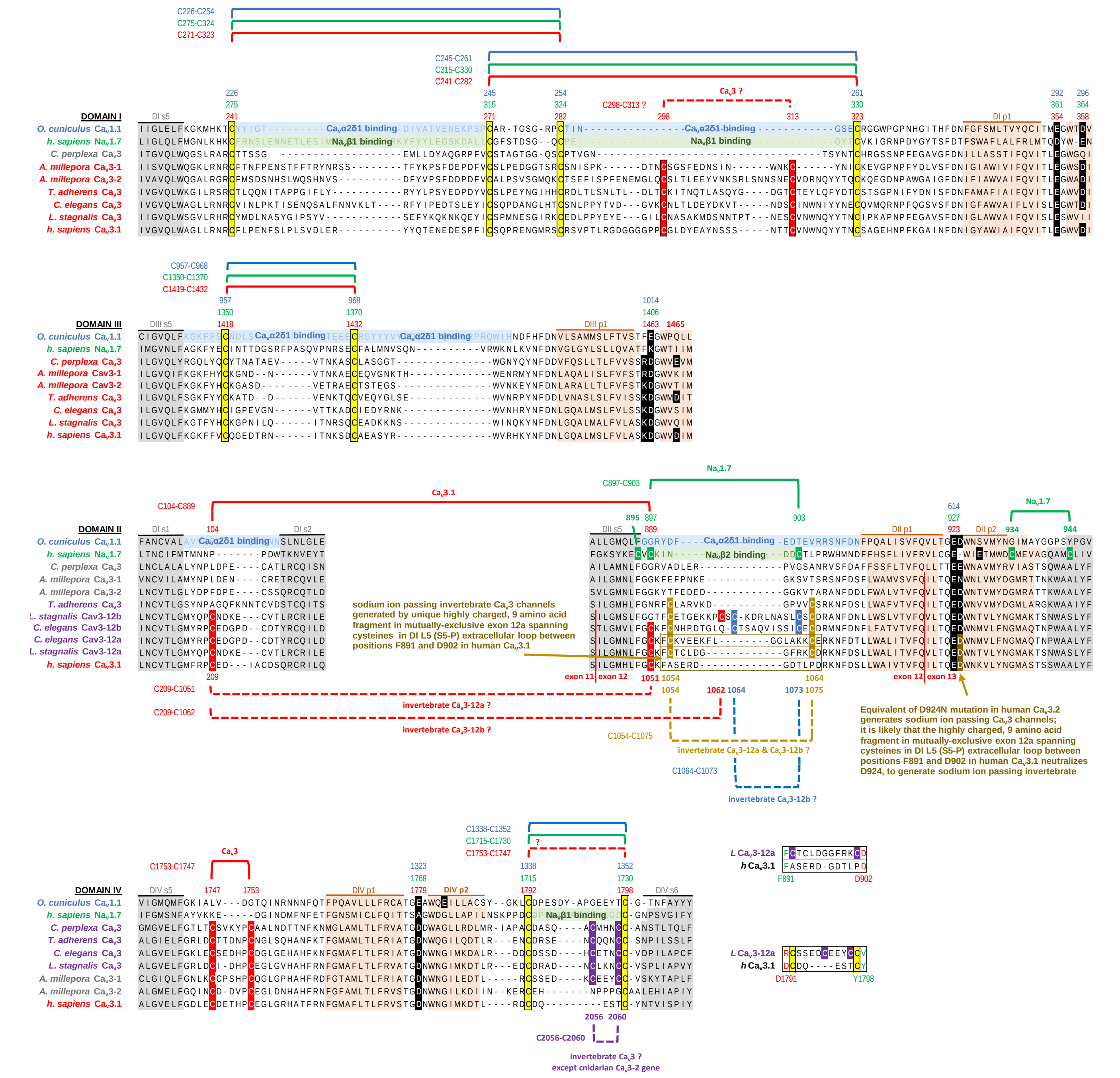

Supplementary Figure 4. Highlighting of the cysteine bonding pairs in amino acid alignments of the extracellular Pore-(P-) loop (s5-P-s6) region spanning DI, DIII, DII and DIV of rabbit $Ca_v1.1$ channel (blue colored pairs), human $Na_v1.7$ channel (green colored pairs) and human $Ca_v3.1$ channel (red colored pairs, including with DII s1-S2 loop), and putative location of additional intra-domain cysteine bonding pairs, in non-vertebrate Ca_v3 channels (purple colored pairs).
Similarly-placed cysteines (yellow colored residues) generate a 2-1-1 framework of shared cysteine pairings amongst all Ca^{2+} (Ca_v1/Ca_v3) and Na^+ (Na_v1) channels. Ca_v3 channels possess additional cysteine pairings above this 2-1-1 configuration within D1L5, D4L5, and between D2 s1-S2 and D2L5 (red colored residues). Some Na_v1 channels (green colored residues) can possess additional cysteine pairing in D2L5 and D2L6. The non-vertebrate Ca_v3 channels possess additional cysteines in D2L5 and D4L6. The extra cysteine loop (C1054-C1064, brown color) contained in exon 12a of invertebrate LCa_v3 channels is modeled to neutralized the equivalent of D924N ($Ca_v3.1$) or D975N ($Ca_v3.2$) in the pore-selectivity filter to generate high Na^+ current passing Ca_v3 channels. A unique cysteine pair in D2L5 of Ca_v3-12b channels is illustrated as light blue residues. Amino acids in extracellular loops involved in the binding of accessory subunits are illustrated $Ca_v\beta1$, $Ca_v\alpha_2\delta_1$ (light blue color): $Na_v\beta1$, Na_v $\beta2$ (light green color). Non-vertebrate Ca_v3 channel sequences: *Choanoeca perplexa* (choanoflagellate), *Acropora millepora* (cnidarian), Trichoplax adherens (placozoan), *Caenorhabiditis elegans* (nematode), *Lymnaea stagnalis* (mollusk). The multiple alignments in the figure were created using MUltiple Sequence Comparison by Log- Expectation (MUSCLE) at website: https://www.ebi.ac.uk/Tools/msa/muscle/ [25].

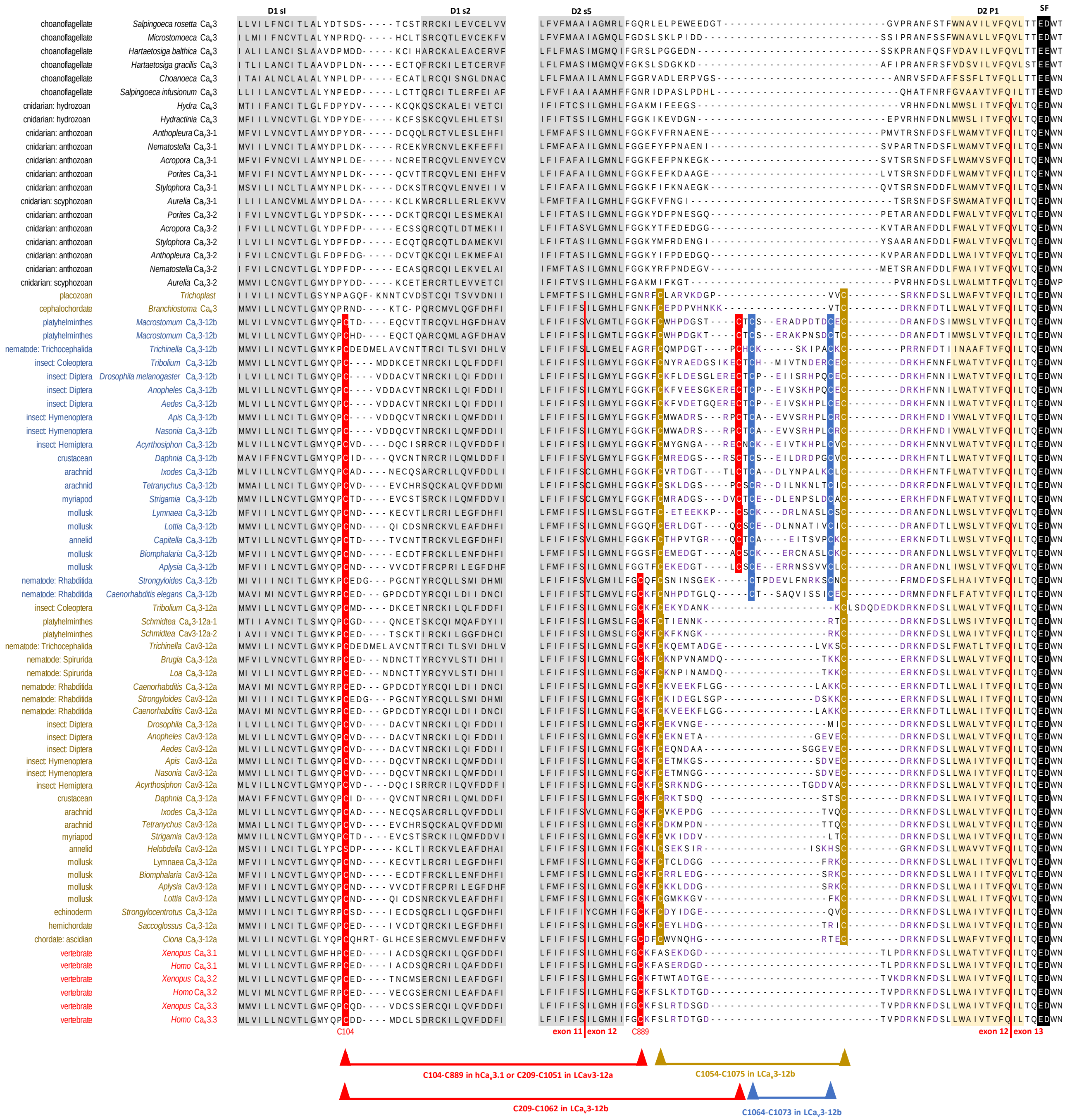

Supplementary Figure 5. Highlighting of the possible evolution of intra-domain cysteine bonding pairs within extracellular DII L5 pore loop (S5-P) and inter-domain cysteine pairing between DII L5 and s1-s2 extracellular loop in the amino acid alignments of seventy-six Ca$_v$3 channels from basal choanoflagellates to human homologs. Vertebrate channels possess a single cysteine pairing C104-C889 (red color) between DII s1-s2 and DII L5 in high resolution structure of Ca$_v$3.1 (**Zhao et al.** (2019) Nature **576**: 492-497) (20). Similarly placed cysteine pairing (C209-C1051, red color) as vertebrate C104-C889 is found in highly sodium ion permeable Ca$_v$3 channels, LCa$_v$3-12a. LCa$_v$3-12a also possesses novel cysteine pair (C1054-C1075, brown color). Novel cysteine pairing C1054-C1075 in LCa$_v$3-12a is modelled to bring charged residues (purple color) in proximity to the pore selectivity filter (SF, black residues). *Trichoplax* and *Branchiostoma* Ca$_v$3 channels lack the equivalent C104-C889 cysteine pairing present in vertebrate Ca$_v$3.1, but does possess the novel cysteine pairing of non-vertebrate LCav3-12a (C1054-C1075, brown color). In the more calcium permeable Cav3 channels containing exon 12b, there are always a novel cysteine pair (C1064-C1073, blue color), in addition to an alternative positioning of the cysteine (C1062, red color) in the DII L5 extracellular loop which is known to bridge to C104, in vertebrate Ca$_v$3.1 channels. Basal species such as single-cell choanoflagellate and cnidarian Ca$_v$3 channels do not possess the splice site to generate mutually-exclusive splicing of exon 12, nor do they possess equivalent cysteines in Domain I s1-s2 and DII L5 extracellular loops. The multiple alignments in the figure were created using MUltiple Sequence Comparison by Log- Expectation (MUSCLE) at website: https://www.ebi.ac.uk/Tools/msa/muscle/ [25].

D4L6 extracellular loops in Ca_v3 T-type channels possess 2 cysteines (cnidarians and vertebrates), or 4 cysteines (cnidarians and non-vertebrates)

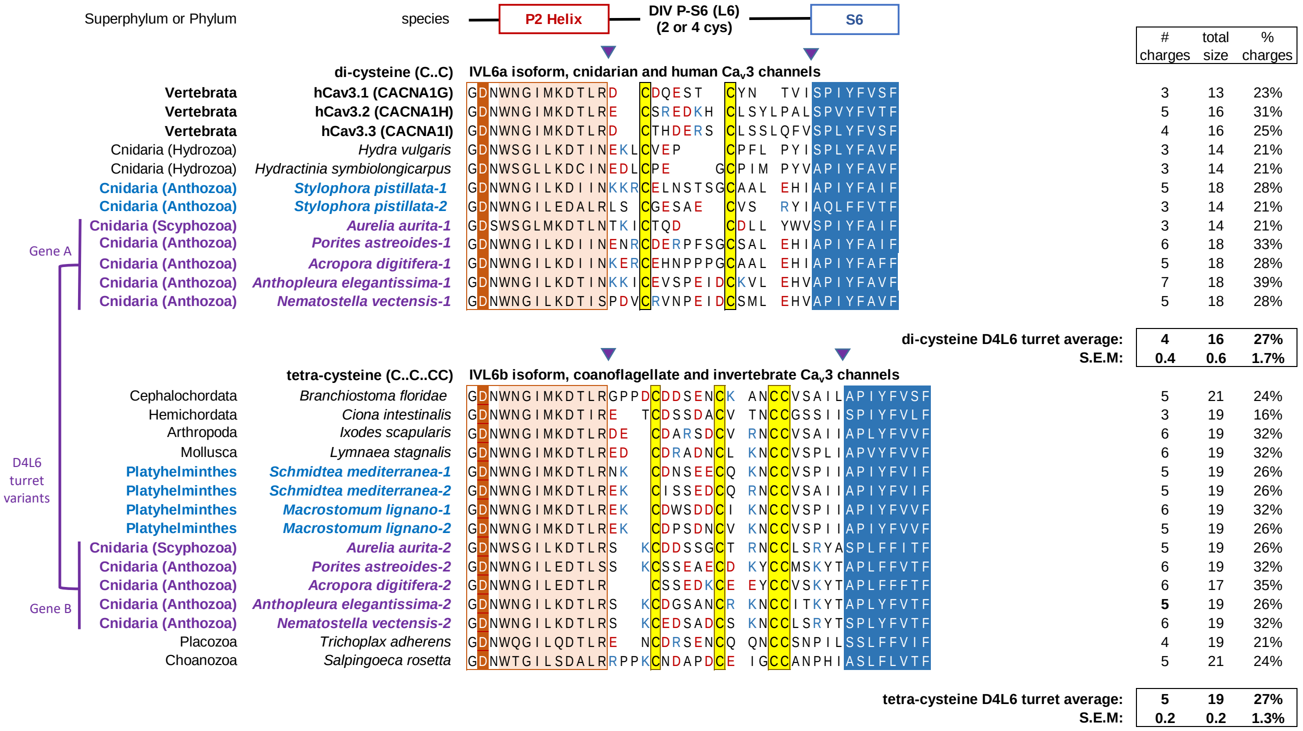

Supplementary Figure 6. Alignment of D4L6 extracellular loops in Ca_v3 T-type channels. Extracellular loops sequences between the P2 (Pore 2) helix (**light orange**) after the selectivity filter (**dark orange**) and transmembrane helix 6 (**dark blue**) in Domain IV of Ca_v3 T-type channels. Pattern of cysteines (**yellow residues**) can be two (cnidarians, vertebrates) or four (non-cnidarian invertebrates. The indicated species containing the same or alternative extracellular loops are labelled in **light blue color names** or **purple colored names**. Note the high density of charged amino acid residues (**red and blue colored residues**) in D2L5 extracellular loops, which would play a role in attracting and binding of ions within the external scaffold above the re-entrant pore. ~1/4 to 1/3 of all residues in the D4L6 extracellular loop are positively or negatively-charged amino acid residues as indicated. The multiple alignments in the figure were created using MUltiple Sequence Comparison by Log- Expectation (MUSCLE) at website: https://www.ebi.ac.uk/Tools/msa/muscle/ [25].

Peak current sizes of representative current traces in solutions containing $\Delta[Ca]_{ex}$ in presence of $[Na]_{ex}$
For snail LCa_v3 channels -/+ containing extracellular D2L5/ D4L6 extracellular loops from human $Ca_v3.2$

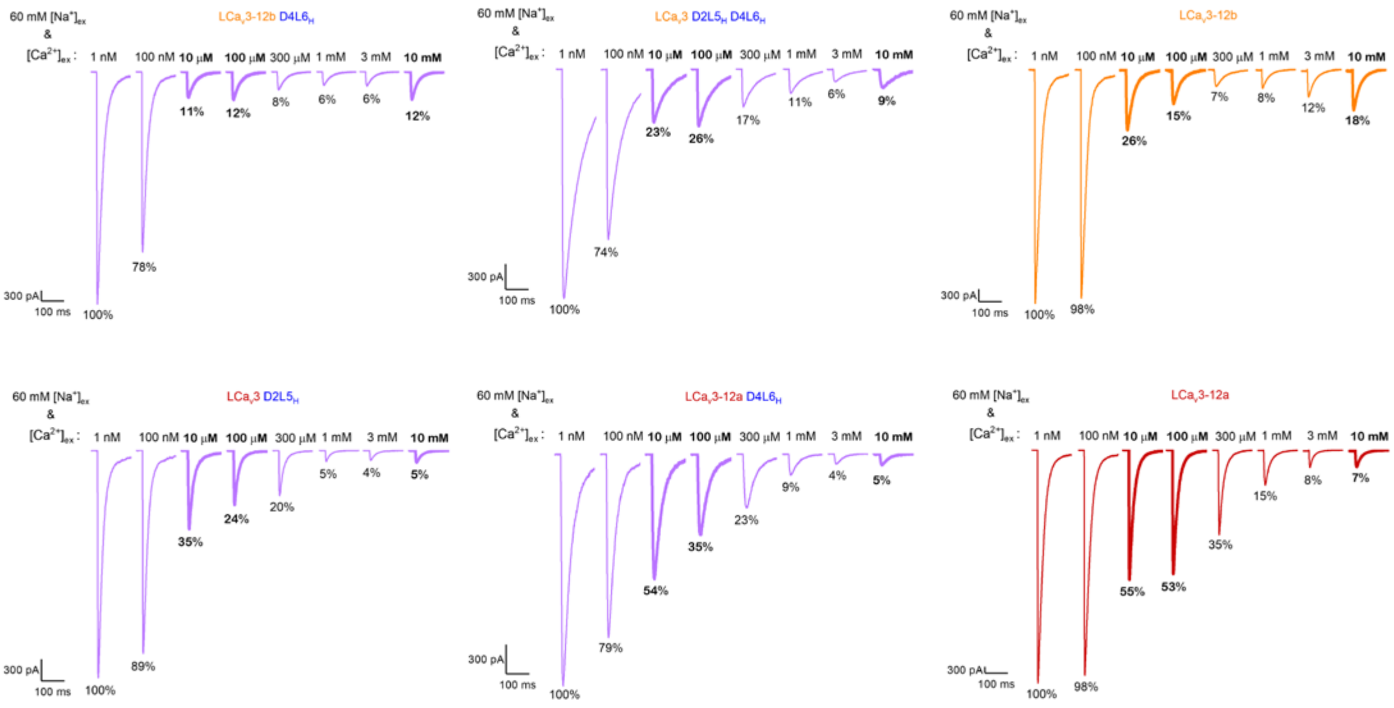

Supplementary Figure 7. D2L5 and D4L6 extracellular loops regulate the degree of Ca^{2+} block of the Na^+ current and the fold increase in relative peak Ca^{2+} current size when external Ca^{2+} rises from 10 µM $[Ca^{2+}]$ex to the physiological (mM) range. (A) Sample traces of peak inward currents for wild type snail LCa_v3-12a and LCa_v3-12b channels, and chimeric snail LCa_v3 channels with D2L5 and D2L6 extracellular loops from human $Ca_v3.2$. Particular current tracings are highlighted because they reflect the key external Ca^{2+} concentrations to assess the degree of Ca^{2+} block of the Na^+ current (10 µM, 100 µM), and relative Ca^{2+} permeability in the physiological range (10 mM). (B, left panel) Normalized peak current sizes in response to increasing concentrations of $[Ca^{2+}]_{ex}$ from 10-9 to 10-2 M in presence 60 mM $[Na^+]_{ex}$. (B, middle panel) Bar graphs of the normalized peak current blockade at 10 µM $[Ca^{2+}]_{ex}$, the maximally effective blocking Ca^{2+} concentration for human Ca_v3 channels (i.e. bottom of "U" shaped curve in A.). (B, right panel) Bar graphs of the fold change in normalized peak currents from 10 µM to 10 mM $[Ca^{2+}]_{ex}$. Graphs illustrate mean ± s.e.m. with replicates (n) illustrated as grey diamonds. Data to generate graphs were compared in a parametric one way ANOVA with a Tukey post hoc analyses to test for statistical significances. Statistical significances are tabulated in Supplementary Tables 8 and 9 for (B, middle panel) and (B, right panel). Data are significant ($p<0.01$), unless stated, where n.s. = non-significant. Data for LCa_v3-12b, LCa_v3-12a and $Ca_v3.1$ in this figure are reproduced integrally from Senatore et al. 2014 (9). Color coding of differing Ca_v3 channels: $Ca_v3.2$ (dark blue), LCa_v3-12b (orange), LCa_v3-12a (red), LCa_v3 Δcys mutants (striped orange or red bars), snail LCa_v3 or human $Ca_v3.2$ channels with chimeric extracellular loops (light purple). Data in this figure were analyzed in OriginPro 2018 (64-bit) SR1 b9.5.1.195.

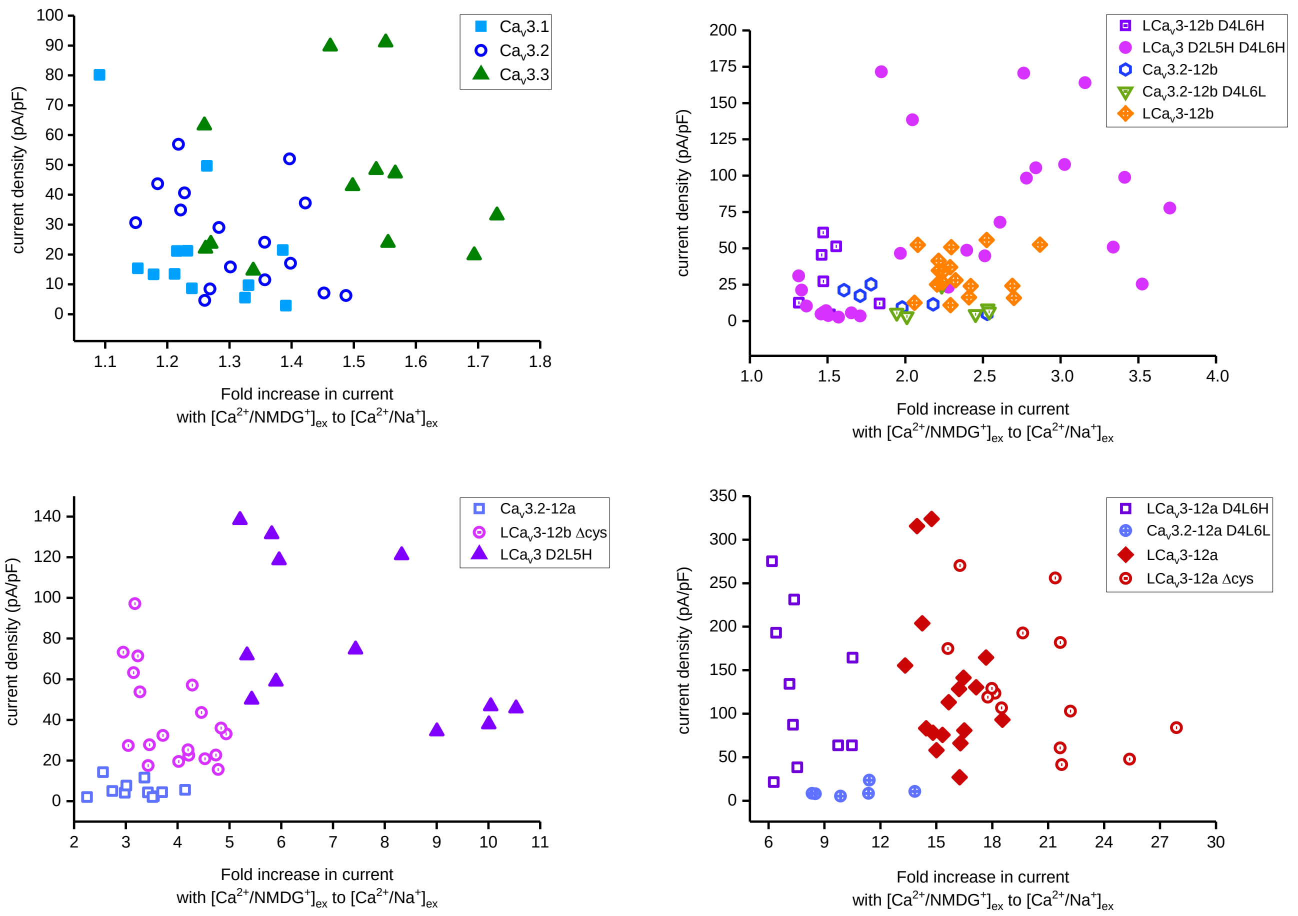

Supplementary Figure 8. Scatter plots illustrate that the fold increase in ionic current due to extracellular sodium ions is not correlated with the current density of expressed Ca_v3 T-type channels transfected in HEK-293T cells. Illustrated are the current densities of sample HEK cells transfected with one of either five wild type Cav3 T-type channels, three mammalian isoforms ($Ca_v3.1$, $Ca_v3.2$ and $Ca_v3.3$) or two snail isoforms (LCav3-12a, LCav3-12b), or ten chimeric channels ($Ca_v3.2$ or LCa_v3) containing differing snail or mammalian D2L5 and/or D4L6 extracellular loops. The current densities of HEK cells containing expressed channels are highly variable, and does not correlate to the fold increase in ionic current due to increase in Na+ current passing capabilities. Data in this figure were analyzed in OriginPro 2018 (64-bit) SR1 b9.5.1.195.

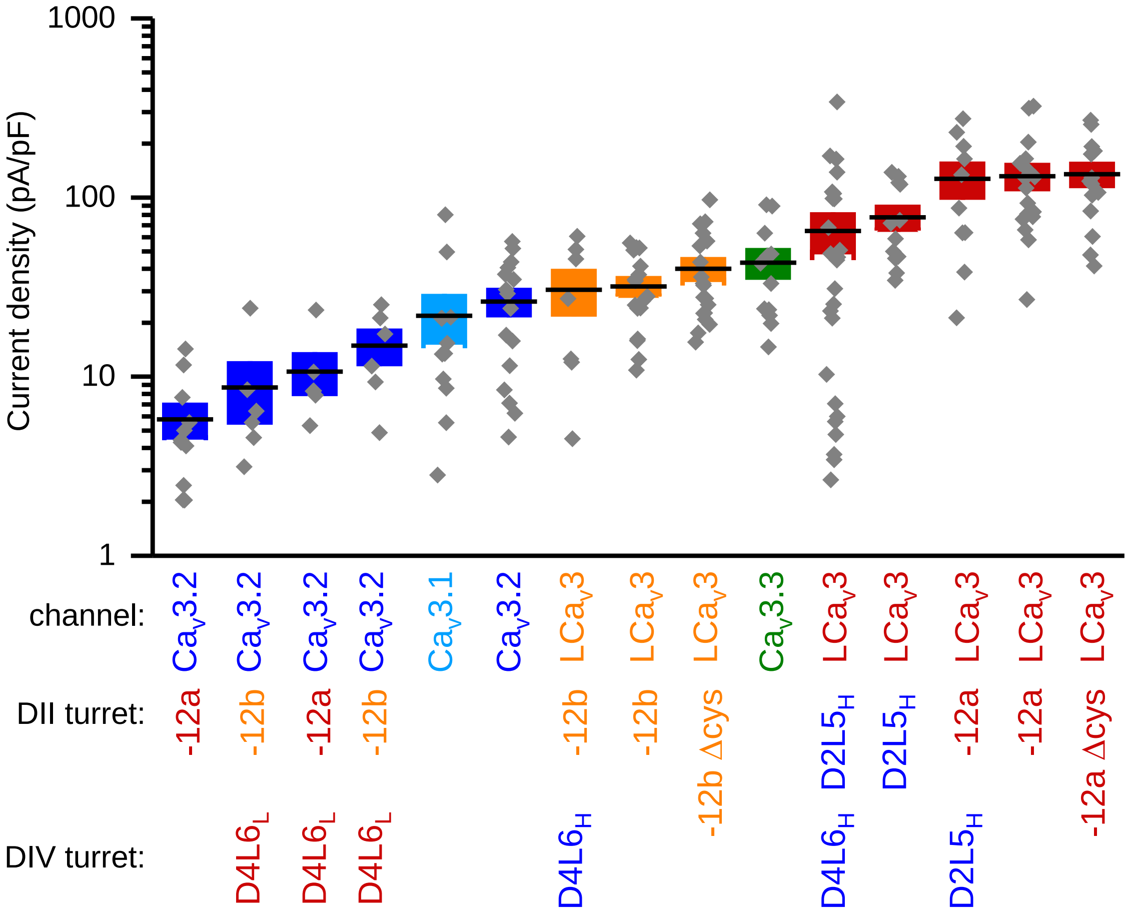

Supplementary Figure 9. The poorest expressing Ca$_v$3 T-type channels transfected in HEK293T cells are the chimeric variants of mammalian Ca$_v$3.2 or snail LCa$_v$3 channels containing D2L5 and/or D4L6 extracellular loops. Box (log) plot illustrating the mean (black line) ± s.e.m. (top and bottom of plot) and individual replicates (grey diamonds) of the five wild-type Ca$_v$3 T-type channels (Ca$_v$3.1, Ca$_v$3.2, Ca$_v$3.3, LCa$_v$3-12a, LCa$_v$3-12b) plus ten chimeric mammalian Ca$_v$3.2 channels or snail LCa$_v$-12a / LCa$_v$3-12b channels containing swapped D2L5 and/or D4L6 extracellular loops. The T-type channels are ordered from lowest to highest current densities (pA/pF) recorded in HEK-293T cells in a log plot. Presence of chimeric D2L5 and/or D4L6 extracellular loops in mammalian Ca$_v$3.2 channels reduces the membrane expression of recordable T-type currents in HEK-293T cells. Data in this figure were analyzed in OriginPro 2018 (64-bit) SR1 b9.5.1.195.

Supplementary Table 1: Values for biophysical parameters for Δ cysteine mutants of LCa$_v$3-12a and LCa$_v$3-12b and the statistical significance comparisons between wild-type and Δ cysteine mutants forms (see data in Fig. 1)[#^]

	LCa$_v$3 12a-Δcys	n	LCa$_v$3 12b-Δcys	n	12a-Δcys 12b-Δcys	12a-Δcys 12a	12b-Δcys 12b	12a-Δcys 12b	12b-Δcys 12a
**Activation**									
$V_{1/2}$	-57.35 ± 0.84	16	-55.86 ± 0.51	21	n.s.	**	n.s.	**	n.s.
K	3.31 ± 0.093	16	3.25 ± 0.096	21	n.s.	**	**	**	**
Peak of IV	- 40 mV	16	-40 mV	21					
**Inactivation**									
$V_{1/2}$	-71.62 ± 0.78	16	-66.50 ± 0.57	12	**	n.s.	**	n.s.	n.s.
K	3.30 ± 0.068	16	2.97 ± 0.06	12	**	*	n.s.	**	**
**Activation kinetics (Time to peak)**									
-55 mV (ms)	12.72 ± 0.73	15	14.46 ± 0.78	21	n.s.	**	**	**	**
-10 mV (ms)	2.19 ± 0.077	15	2.4 ± 0.076	21	n.s.	**	**	**	**
**Inactivation kinetics (τ)**									
-55 mV (ms)	31.14 ± 2.49	13	57.03 ± 3.51	18	**	n.s.	**	*	**
-10 mV (ms)	11.41 ± 0.28	13	11.6 ± 0.37	18	n.s.	**	**	**	**
**Deactivation** (τ)									
-100 mV (ms)	1.02 ± 0.050	16	0.55 ± 0.030	11	**	**	**	**	**
-60 mV (ms)	7.17 ± 0.37	16	3.40 ± 0.44	11	**	n.s.	**	**	**
**Recovery from Inactivation**									
% recovery at 0.25s	4.0 ± 0.0040	13	6.36 ± 0.0071	8	**	**	n.s.	n.s.	*
% recovery at 5s	80.85 ± 0.013	13	58.20 ± 0.010	8	**	**	*	**	**
$T_{0.5}$ (ms)	1481.19 ± 64.60	13	2492.6 ± 85.75	8	**	**	*	**	**

[#]One-way ANOVA combined with a Tukey *post hoc* test. *p<0.05, **p<0.01, n.s., not significant
^Data in this Table were analyzed in OriginPro 2018 (64-bit) SR1 b9.5.1.195.

A

Supplementary Table 2: Values for bi-ionic reversal potentials and relative (P_{Ca}/P_x) permeabilities (for Fig. 3)^

reversal potentials :	Li	s.e.m.	n	Na	s.e.m.	n	K	s.e.m.	n	Cs	s.e.m.	n
Erev Cav3.1	21.74	0.61	7	28.82	0.87	6	41.41	0.63	6	42.96	0.67	6
Erev Cav3.2	22.29	0.56	6	29.06	0.53	6	41.02	0.72	6	42.60	0.56	6
Erev Cav3.3	16.39	0.28	6	23.28	0.40	6	38.73	0.36	6	40.35	0.45	6
Erev LCav3-12b	13.21	0.58	6	19.76	0.50	7	28.31	0.41	8	33.79	0.51	8
Erev LCav3-12a	9.40	0.33	9	15.31	0.30	6	21.86	0.49	13	31.42	0.43	6
Erev LCav3-12b (Δcys)	3.41	0.56	14	10.37	0.25	11	12.27	0.43	15	16.59	0.28	13
Erev LCav3-12a (Δcys)	-0.75	0.82	12	4.68	0.74	12	6.12	0.39	11	16.83	0.76	11

relative permeabilities :	P_{Ca}/P_{Li}	s.e.m.	n	P_{Ca}/P_{na}	s.e.m.	n	P_{Ca}/P_K	s.e.m.	n	P_{Ca}/P_{Cs}	s.e.m.	n
$P_{Ca}/_{Px}$ Cav3.1	48.76	2.01	7	78.79	4.60	6	140.16	12.02	6	189.32	8.32	6
$P_{Ca}/_{Px}$ Cav3.2	50.49	1.89	6	79.66	2.85	6	172.49	9.12	6	184.33	9.48	6
$P_{Ca}/_{Px}$ Cav3.3	34.25	0.60	6	53.85	1.47	6	151.00	6.90	6	155.86	3.94	6
$P_{Ca}/_{Px}$ LCav3-12b	28.03	1.05	6	42.75	1.48	7	78.04	2.81	8	75.65	2.16	8
$P_{Ca}/_{Px}$ LCav3-12a	22.04	0.44	9	31.95	0.61	6	50.25	1.56	13	49.22	1.58	6
Erev LCav3-12b (Δcys)	15.40	0.50	14	23.40	0.36	11	26.21	0.71	15	26.46	0.70	13
Erev LCav3-12a (Δcys)	12.12	0.55	12	16.70	0.74	12	18.85	0.66	11	18.05	0.43	11

B

Supplementary Table 3: Statistic significance testing for relative (P_{Ca}/P_x) permeabilities (for Fig. 3)#^

Relative permeability (different Ca_v3 channel, same monovalent ion comparison)

	LCa_v-12a						LCa_v3-12a Δcys					LCa_v3-12b				LCa_v3-12b Δcys			Ca_v3.1		Ca_v 3.2
	3.1	3.2	3.3	12b	12b Δcys	12a Δcys	3.1	3.2	3.3	12b	12b Δcys	3.1	3.2	3.3	12b Δcys	3.1	3.2	3.3	3.2	3.3	3.3
Li^+	**	**	**	**	**	**	**	**	**	**	n.s.	**	**	**	**	**	**	**	n.s.	**	**
Na^+	**	**	**	**	**	**	**	**	**	**	**	**	**	**	**	**	**	**	n.s.	**	**
K^+	**	**	**	**	**	**	**	**	**	**	n.s.	**	**	**	**	**	**	**	n.s.	**	**
Cs^+	**	**	**	n.s.	**	**	**	**	**	**	n.s.	**	**	**	**	**	**	**	n.s.	**	**

#One-way ANOVA combined with a Tukey *post hoc* test. *p<0.05, **p<0.01, n.s., not significant

^Data in this Table were analyzed in OriginPro 2018 (64-bit) SR1 b9.5.1.195.

A

Supplementary Table 4: Statistical comparison of fold increase in I_{peak} with Na_{ex} of D2L5/D4L6 extracellular loop chimeras in snail LCa$_v$3 channel background (see Figs. 2c, 9a)[#]^

	Ca$_v$3.1	Ca$_v$3.2	Ca$_v$3.3	LCa$_v$3 12b D4L6$_H$	LCa$_v$3 D2L5$_H$ D4L6$_H$	LCa$_v$3 12b	LCa$_v$3 D2L5$_H$	LCa$_v$3 12a D4L6$_H$	LCa$_v$3 12a
Ca$_v$3.1									
Ca$_v$3.2	n.s.								
Ca$_v$3.3	n.s.	n.s.							
LCa$_v$3 12b D4L6$_H$	n.s.	n.s.	n.s.						
LCa$_v$3 D2L5$_H$ D4L6$_H$	n.s.	n.s.	n.s.	n.s.					
LCa$_v$3 12b	n.s.	n.s.	n.s.	n.s.	n.s.				
LCa$_v$3 D2L5$_H$	**	**	**	**	**	**			
LCa$_v$3 12a D4L6$_H$	**	**	**	**	**	**	n.s.		
LCa$_v$3 12a	**	**	**	**	**	**	**	**	

B

Supplementary Table 5: Statistical comparison of fold increase in I_{peak} with Na_{ex} of D2L5/D4L6 extracellular loop chimeras in mammalian Ca$_v$3.2 channel background (see Figs. 2c, 9b)[#]^

	Ca$_v$3.1	Ca$_v$3.2	Ca$_v$3.3	Ca$_v$3.2 12b	Ca$_v$3.2 12b D4L6$_L$	LCa$_v$3 12b	Ca$_v$3.2 12a	Ca$_v$3.2 12a D4L6$_L$	LCa$_v$3 12a
Ca$_v$3.1									
Ca$_v$3.2	n.s.								
Ca$_v$3.3	n.s.	n.s.							
Ca$_v$3.2 12b	n.s.	n.s.	n.s.						
Ca$_v$3.2 12b D4L6$_L$	n.s.	n.s.	n.s.	n.s.					
LCa$_v$3 12b	**	**	n.s.	n.s.	n.s.				
Ca$_v$3.2 12a	**	**	**	n.s.	n.s.	n.s.			
Ca$_v$3.2 12a D4L6$_L$	**	**	**	**	**	**	**		
LCa$_v$3 12a	**	**	**	**	**	**	**	**	

[#]One-way ANOVA combined with a Tukey *post hoc* test. *p<0.05, **p<0.01, n.s., not significant

^Data in this Table were analyzed in OriginPro 2018 (64-bit) SR1 b9.5.1.195.

A

Supplementary Table 6: Statistical comparison of reversal potentials in bi-ionic solution [Ca^{2+}]$_{ex}$ & [Li$^+$]$_{in}$ (see Fig. 3, 10a)#^

	Ca$_v$3.1	Ca$_v$3.2	Ca$_v$3.3	LCa$_v$3 12b D4L6$_H$	LCa$_v$3 D2L5$_H$ D4L6$_H$	LCa$_v$3 12b	LCa$_v$3 12a D4L6$_H$	LCa$_v$3 D2L5$_H$	LCa$_v$3 12a	LCa$_v$3 12b Δcys	LCa$_v$3 12a Δcys
Ca$_v$3.1											
Ca$_v$3.2	n.s.										
Ca$_v$3.3	**	**									
LCa$_v$3 12b D4L6$_H$	**	**	n.s.								
LCa$_v$3 D2L5$_H$ D4L6$_H$	**	**	n.s.	n.s.							
LCa$_v$3 12b	**	**	n.s.	n.s.	n.s.						
LCa$_v$3 12a D4L6$_H$	**	**	**	**	**	n.s.					
LCa$_v$3 D2L5$_H$	**	**	**	**	**	n.s.	n.s.				
LCa$_v$3 12a	**	**	**	**	**	**	n.s.	n.s.			
LCa$_v$3 12b Δcys	**	**	**	**	**	**	**	**	**		
LCa$_v$3 12a Δcys	**	**	**	**	**	**	**	**	**	**	

B

Supplementary Table 7: Statistical comparison of reversal potentials in bi-ionic solution [Ca^{2+}]$_{ex}$ & [Na$^+$]$_{in}$ (see Fig. 3, 10b)#^

	Ca$_v$3.1	Ca$_v$3.2	Ca$_v$3.3	LCa$_v$3 12b D4L6$_H$	LCa$_v$3 D2L5$_H$ D4L6$_H$	LCa$_v$3 12b	LCa$_v$3 12a D4L6$_H$	LCa$_v$3 D2L5$_H$	LCa$_v$3 12a	LCa$_v$3 12b Δcys	LCa$_v$3 12a Δcys
Ca$_v$3.1											
Ca$_v$3.2	n.s.										
Ca$_v$3.3	**	**									
LCa$_v$3 12b D4L6$_H$	n.s.	n.s.	n.s.								
LCa$_v$3 D2L5$_H$ D4L6$_H$	n.s.	n.s.	n.s.	n.s.							
LCa$_v$3 12b	**	**	**	**	**						
LCa$_v$3 12a D4L6$_H$	**	**	**	**	**	**					
LCa$_v$3 D2L5$_H$	**	**	**	**	**	**	n.s.				
LCa$_v$3 12a	**	**	**	**	**	**	n.s.	n.s.			
LCa$_v$3 12b Δcys	**	**	**	**	**	**	**	**	**		
LCa$_v$3 12a Δcys	**	**	**	**	**	**	**	**	**	**	

#One-way ANOVA combined with a Tukey *post hoc* test. *p<0.05, **p<0.01, n.s., not significant
^Data in this Table were analyzed in OriginPro 2018 (64-bit) SR1 b9.5.1.195.

A

Supplementary Table 8: Statistical comparison of relative % block of peak Na^+ current in presence of 10 mM $[Ca^{2+}]_{ex}$ (see Figs. 4, 11)#^

	Ca$_v$3.1	Ca$_v$3.2	Ca$_v$3.3	LCa$_v$3 12b D4L6$_H$	LCa$_v$3 D2L5$_H$ D4L6$_H$	LCa$_v$3 12b	LCa$_v$3 12b Δcys	LCa$_v$3 D2L5$_H$	LCa$_v$3 12a D4L6$_H$	LCa$_v$3 12a	LCa$_v$3 12a Δcys
Ca$_v$3.1											
Ca$_v$3.2	n.s.										
Ca$_v$3.3	n.s.	n.s.									
LCa$_v$3 12b D4L6$_H$	n.s.	n.s.	n.s.								
LCa$_v$3 D2L5$_H$ D4L6$_H$	**	**	**	**							
LCa$_v$3 12b	**	**	**	**	n.s.						
LCa$_v$3 12b Δcys	**	**	**	**	n.s.	n.s.					
LCa$_v$3 D2L5$_H$	**	**	**	**	**	**	n.s.				
LCa$_v$3 12a D4L6$_H$	**	**	**	**	**	**	**	**			
LCa$_v$3 12a	**	**	**	**	**	**	**	**	n.s.		
LCa$_v$3 12a Δcys	**	**	**	**	**	**	**	**	**	**	

B

Supplementary Table 9: Statistical comparison of fold increase in relative peak current from 10 μM to 10 mM $[Ca]_{ex}$ in presence of $[Na]_{ex}$ (see Figs. 4, 11)#^

	Ca$_v$3.1	Ca$_v$3.2	Ca$_v$3.3	LCa$_v$3 12b D4L6$_H$	LCa$_v$3 D2L5$_H$ D4L6$_H$	LCa$_v$3 12b	LCa$_v$3 12b Δcys	LCa$_v$3 D2L5$_H$	LCa$_v$3 12a D4L6$_H$	LCa$_v$3 12a	LCa$_v$3 12a Δcys
Ca$_v$3.1											
Ca$_v$3.2	n.s.										
Ca$_v$3.3	**	**									
LCa$_v$3 12b D4L6$_H$	**	**	**								
LCa$_v$3 D2L5$_H$ D4L6$_H$	**	**	**	n.s.							
LCa$_v$3 12b	**	**	**	n.s.	n.s.						
LCa$_v$3 12b Δcys	**	**	**	n.s.	n.s.	n.s.					
LCa$_v$3 D2L5$_H$	**	**	**	**	**	**	**				
LCa$_v$3 12a D4L6$_H$	**	**	**	**	**	**	**	n.s.			
LCa$_v$3 12a	**	**	**	**	**	**	**	n.s.	n.s.		
LCa$_v$3 12a Δcys	**	**	**	**	**	**	**	**	n.s.	**	

#One-way ANOVA combined with a Tukey *post hoc* test. *p<0.05, **p<0.01, n.s., not significant

^Data in this Table were analyzed in OriginPro 2018 (64-bit) SR1 b9.5.1.195.
